# M2 polarization of macrophage protects the lung cancer cells from cold atmospheric plasma *via* alleviating endoplasmic reticulum stress

**DOI:** 10.1038/s41420-025-02775-4

**Published:** 2025-10-27

**Authors:** Yue Feng, Shengjie Peng, Lele Zhao, Renyi Jiang, Chunhua Tan, K. N. Yu, Lianjun Chen, Quan Li, Ye Zhao, Wei Han

**Affiliations:** 1 Hefei Cancer Hospital of CAS, Institute of Health and Medical Technology, Hefei Institutes of Physical Science, Chinese Academy of Sciences, Hefei, PR China; 2 Teaching and Research Section of Nuclear Medicine, School of Basic Medical Sciences, Anhui Medical University, Hefei, PR China; 3 Department of Physics, City University of Hong Kong, Hong Kong, PR China; 4 School of Biology, Food and Environment, Hefei University, Hefei, PR China; 5 Department of Radiation Medicine, School of Public Health and Management, Wenzhou Medical University, Wenzhou, PR China; 6 Collaborative Innovation Center of Radiation Medicine of Jiangsu Higher Education Institutions and School for Radiological and Interdisciplinary Sciences (RAD-X), Soochow University, Suzhou, PR China

**Keywords:** Apoptosis, Cancer immunotherapy

## Abstract

Although cold atmospheric plasma (CAP) has been proved to kill various kinds of tumor cells effectively, most previous studies were performed on the in vitro tumor cell model in the absence of tumor microenvironment (TME), resulting in limited insights to its clinical application. Here, we explored the anti-tumor effect of CAP based on a co-culture model of macrophages and lung cancer cells, and it was found that CAP could induce M2 polarization of macrophages and then release IL-10. The released IL-10 activated the STAT1/STAT3 signaling pathway to alleviate CAP-induced endoplasmic reticulum stress in tumor cells, finally resulting in attenuation of programmed death of tumor cells after CAP exposure. In particular, the presence of macrophages caused the reduction of GSDME-dependent pyroptosis, which was proved to play an important role in activation of anti-tumor immunity, induced by CAP. Our findings provide evidences to a better understanding of the anti-tumor effect of CAP and insights to promote the clinical application of CAP tumor therapy.

## Introduction

Cold atmospheric plasma (CAP), a gas mixture composed of ions, electrons, photons and free radicals etc., can be generated at room temperature [1, 2]. CAP has been considered a promising technique of tumor therapy for its remarkable anti-cancer effect in vitro and in vivo [3].

CAP contains large amounts of reactive oxygen species (ROS) and reactive nitrogen species (RNS), including hydroxyl radical (•OH), hydrogen peroxide (H_2_O_2_), nitrogen trioxide (NO_3_^•^), nitric oxide (NO^•^) and peroxynitrite (ONOO^−^), which are the bases of its clinical application [4, 5]. The extensive ROS/RNS cause oxidative stress to damage DNA and proteins, and then lead to organelles dysfunction [6]. Moreover, previous studies also revealed that CAP effectively induced diverse cell death types in tumor cells, including apoptosis, autophagy, pyroptosis and ferroptosis [7–9], and the possible mechanisms of these CAP-induced programmed cell death have also been investigated.

Besides the direct effect to tumor cells, it was also reported that CAP could enhance immune cell function and act as a direct immunomodulator to alter the phenotype of immune cells (e.g., differentiation and polarization of macrophages, receptor expression of DC and T cells), and to regulate the secretion of chemokines/cytokines of immune cells [10, 11]. It is speculated that CAP might enhance the anti-tumor effect *via* pro-inflammatory immune response. However, a good understanding of the therapeutic effect of CAP is extremely difficult for clinical applications since the actual tumor microenvironment (TME) is complicated in vivo and the interaction between immune cells and tumor cells might be more intricate than we expect.

Macrophage, the largest proportion of immune cells in TME, is associated with tumor prognosis and drug resistance [12]. It is known that M2 macrophages release anti-inflammatory cytokines, including TGF-β, IL-10, Arginase-I and VEGF [13, 14], to inhibit the immune response of tumor cells and promote the occurrence, progression and metastasis of tumor [15, 16]. Among these cytokines, IL-10 plays a pro-tumor role through regulating a variety of signaling pathways, including ERK1/2, STAT3 and NF-κB, and regulating the expression of related genes [17]. It has been reported that CAP could regulate the polarization of macrophages to affect the immunomodulatory function. Freund et al. reported that CAP promoted the differentiation of monocytes into macrophages, specifically M1 macrophages [18]. In addition, the expression of CD206 in monocyte-derived macrophages was upregulated after CAP treatment, indicating that CAP could also induce M2 macrophages [19, 20]. However, most of the previous studies were mainly based on cultured tumor cells and the potential role of TME was not mentioned.

Our findings demonstrate that CAP treatment could induce M2 polarization of macrophages, trigger IL-10 release, subsequently activate the STAT1/STAT3 signaling pathway to alleviate ER stress caused by CAP, and ultimately suppress the programmed cell death in lung cancer cells, including apoptosis, pyroptosis and autophagy. This study reveals that macrophages in the TME interfere with and even reduce the tumor killing effect of CAP treatment, and clarifies the related molecular mechanisms promoting tumor cell survival, which would provide new insights for the complicated effects of CAP on immune cells and the immune system. These findings will broaden and deepen the understandings of multifaceted biological effects of CAP, contribute to revealing potential problems during CAP treatment in vivo, and help to improve the clinical application for oncotherapy.

## Results

### Macrophages significantly reduced the killing effect of CAP on tumor cells

**Fig. 1:**
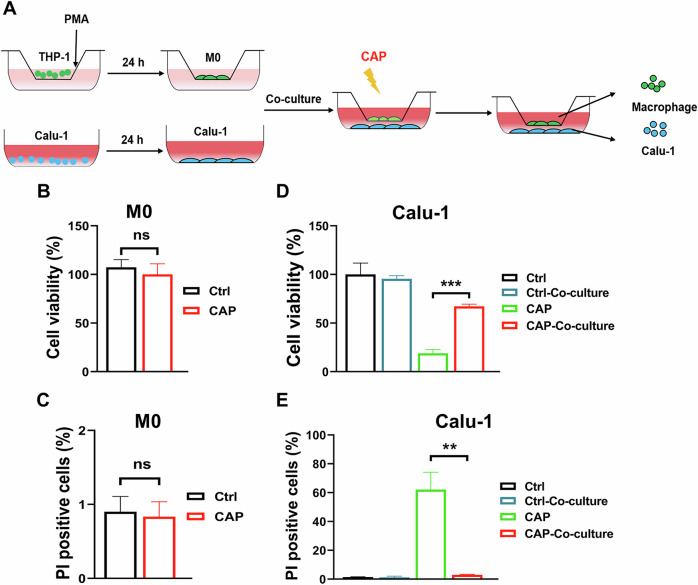
Macrophages significantly reduced the killing effect of CAP on tumor cells. **A** Scheme of lung cancer cells and macrophages co-culture system. **B** Viability of M0 cells treated with CAP (30 s). **C** Induction of cell death in M0 macrophages after CAP (30 s) treatment. **D** Viability of Calu-1 cells treated with CAP (30 s). **E** Induction of cell death in Calu-1 cells after CAP (30 s). ns no significance, **: *p* < 0.01.

Firstly, the killing effect of CAP on separately cultured M0 macrophages (differentiated from THP-1 cells) and lung cancer cells was determined with CCK-8 and PI staining at 24 h after CAP treatment. As shown in Fig. 1, CAP treatment (30 s) did not decrease the viability of M0 cells and induce cell death significantly (Fig. 1B, C). However, the same dose of CAP treatment decreased the viability of Calu-1 cells into 18.9 ± 3.8% of the control (Fig. 1D) and induced 62.1 ± 11.9% PI positive cells (dead cells) (Fig. 1E).

However, the presence of macrophages in the co-culture system significantly attenuated the viability decrease of Calu-1 cells after CAP exposure (Fig. 1D). Similarly, cell death determination with PI staining displayed a lower cell death rate of Calu-1 cells when co-cultured with macrophages, compared to CAP-treated alone (Fig. 1E). Consistent trends were also observed in H1299 and H1975 cells (Extended Fig. 1A~D). These results indicate that CAP treatment triggers the secretion of some factor(s) from macrophages, which significantly alleviate the killing effect of CAP on the co-cultured tumor cells.

### M2 polarization of macrophage induced by CAP attenuated the killing effect of CAP on tumor cells

**Fig. 2:**
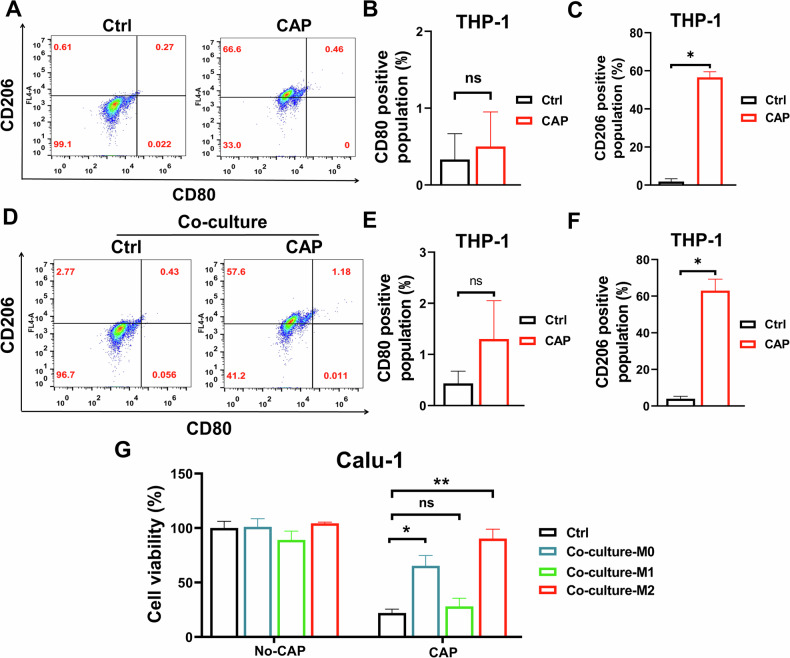
CAP (30 s) induces polarization of M2 macrophages to attenuate the killing effect. **A** Typical results of flow cytometry for detection of macrophage polarization. The fraction of CD80 (**B**) and CD206 (**C**) positive macrophages after CAP treatment. **D** Typical results of flow cytometry for detection of polarization of macrophages co-cultured with Calu-1 cells. The fraction of CD80 (**E**) and CD206 (**F**) positive macrophages, co-cultured with Calu-1 cells, after CAP treatment. **G** Viability of Calu-1 cells co-cultured with M0, M1 or M2 macrophages after CAP treatment. ns no significance, *: *p* < 0.05; **: *p* < 0.01.

It is known that the macrophages polarized to M1 or M2 exhibited entirely different effects. To investigate the effect of CAP on macrophage, we firstly detected the polarization of separately cultured macrophages after CAP treatment (30 s). The results showed that CD206^+^ cells (the marker of M2 macrophage) but not CD80^+^ cells (the marker of M1 macrophage) increased distinctly after CAP treatment (Fig. 2A-C), indicating that CAP induced M2 polarization. Similarly, the polarization of macrophages (co-cultured with Calu-1 cells) also showed the same trend after CAP treatment (Fig. 2D-F).

To determine the effect of M2 polarization on the co-cultured Calu-1 cells, we induced M0 macrophages to M1 or M2 type *via* treating with LPS + IFN-γ or IL-4 + IL-13 for 48 h (Extended Fig. 2B~G), respectively, and then co-cultured with Calu-1 cells for CAP treatment. As showed in Fig. 2G, the viability of Calu-1 cells co-cultured with M1 macrophages significantly decreased after CAP treatment, almost to the same level of separately cultured Calu-1 cells after CAP treatment. However, the viability of Calu-1 cells co-cultured with M2 macrophages was much higher than that of separately cultured Calu-1 cells after the same CAP treatment.

These results indicate that the presence of M2 macrophages in the co-culture system significantly attenuates the killing effect of CAP on tumor cells.

### Macrophages attenuate the killing effect of CAP through IL-10-STAT1/STAT3 pathway

**Fig. 3:**
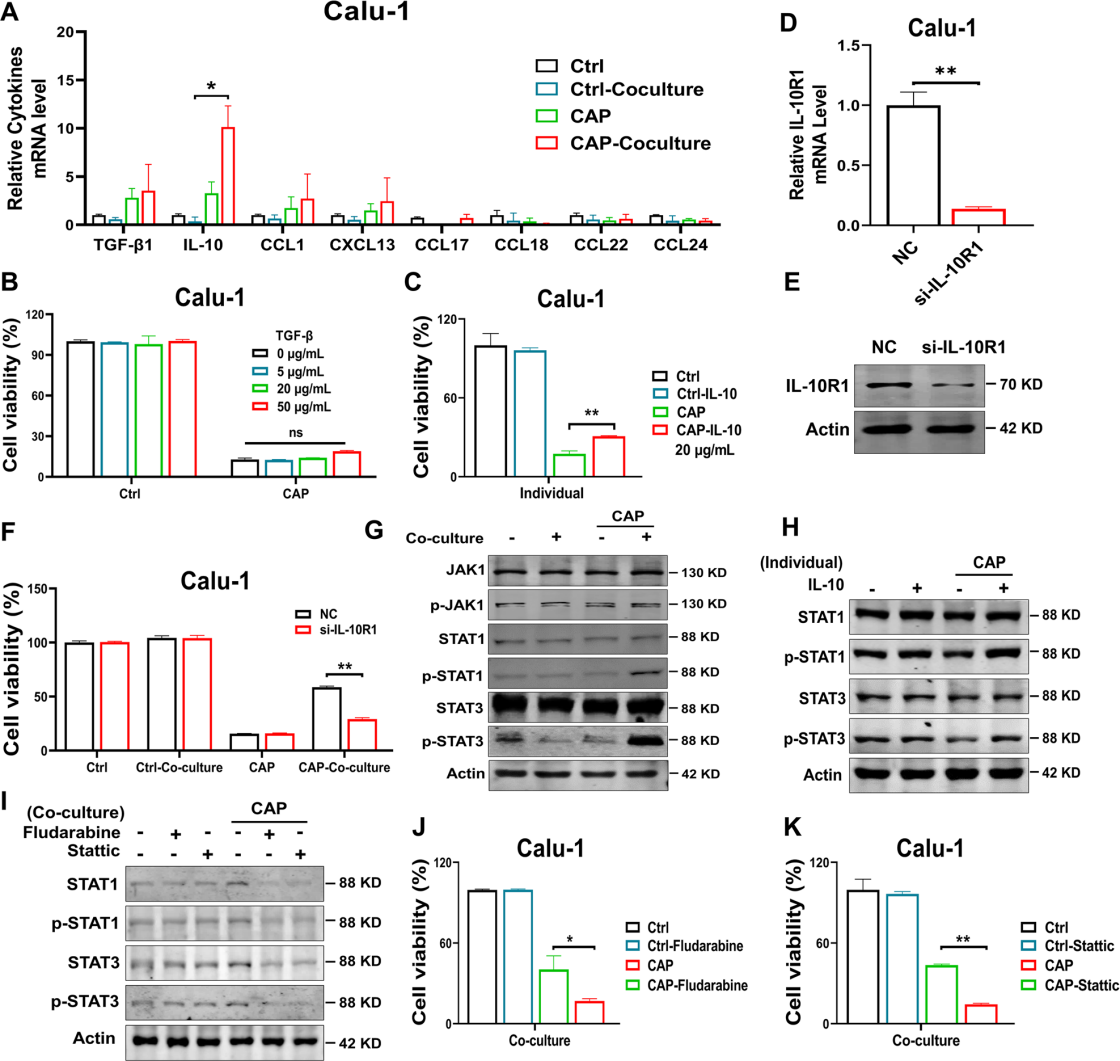
Macrophages attenuate the killing effect of CAP through IL-10-STAT1/STAT3 pathway. **A** Transcription level of cytokines in co-cultured macrophages after CAP treatment. **B, C** Effect of exogenous TGF-β (**B**) or IL-10 (**C**) on the viability of separately cultured Calu-1 cells treated with CAP. **D**, **E** Transcription and protein expression levels of IL-10R1 in Calu-1 cells. **F** Viability of co-cultured Calu-1 cells treated with CAP. **G** Protein expression Calu-1 cells after CAP treatment. **H** Effect of IL-10 (20 μg/mL) on protein expression in separately cultured Calu-1 cells after CAP treatment. **I** Effect of Fludarabine (10 mM) or Stattic (30 mM) on protein expression in co-cultured Calu-1 cells after CAP treatment. **J, K** Effect of Fludarabine (10 mM) (**J**) or Stattic (30 mM) (**K**) on the viability of co-cultured Calu-1 cells after CAP treatment. ns no significance, *: *p* < 0.05; **: *p* < 0.01.

Considering that secretion of cytokines was the main function exerted by macrophages except for phagocytosis [21], we supposed that macrophages treated by CAP treatment might release some cytokines to act on the co-cultured Calu-1 cells rather than physical contact. We detected the transcription level of various cytokines in CAP-treated macrophages with qRT-PCR. Notably, the transcription levels of IL-10 and TGF-β1 were significantly upregulated in the macrophages after CAP treatment (Fig. 3A).

To further elucidate the functional roles of IL-10 or TGF-β1, exogenous IL-10 or TGF-β1 recombinant proteins were used to treat tumor cells for 12 h, respectively, and then the cell viability was detected at 24 h after CAP treatment. Strikingly, while exogenous TGF-β1 treatment (5~50 μg/mL) showed no distinctly effect on Calu-1 cells viability after CAP treatment compared to the control (Fig. 3B), IL-10 (20 μg/mL) pretreatment significantly alleviated the decrease of Calu-1 cell viability caused by CAP treatment (Fig. 3C). Meanwhile, the secretion level of IL-10 from THP-1 cells in co-culture supernatants was significantly higher than control after CAP treatment (Extended Fig. 3A). These results demonstrate that IL-10, but not TGF-β1 protects tumor cells from CAP treatment.

To verify the critical role of IL-10, specific siRNA was used to interfere with the expression of IL-10 receptor 1 (IL-10R1) in Calu-1 cells before co-culture with macrophages for CAP treatment. The effect of IL-10R1 interference was significant (Fig. 3D, E) and the results in Fig. 3F showed that IL-10R1 interference significantly eliminated the protection effect of macrophages, indicating the important role of IL-10 in the macrophages protecting tumor cells against CAP treatment.

Furthermore, to explore the possible mechanism of IL-10 attenuating the killing effect of CAP on tumor cells, the relevant signaling pathways regulated by IL-10 were detected. The results in Fig. 3G showed that the phosphorylation level of JAK1 in the co-cultured Calu-1 cells did not change significantly at 6 h after CAP treatment compared to that in the separately cultured Calu-1 cells. However, the phosphorylation levels of both STAT1 and STAT3 elevated significantly (Fig. 3G). Interestingly, Calu-1 cells pretreated with IL-10 showed a similar phosphorylation trend of STAT1/STAT3 after CAP treatment (Fig. 3H).

To further verify whether the STAT1/STAT3 signaling pathway was involved in macrophages protecting tumor cells from CAP treatment, Fludarabine (STAT1 transcription inhibitor) or Stattic (STAT3 phosphorylation inhibitor) was employed to treat Calu-1 cells. As shown in Fig. 3I–K, treatment with either Fludarabine or Stattic significantly reduced the viability of co-cultured Calu-1 cells after CAP treatment compared to those without inhibitor treatment, indicating that the activation of STAT1/STAT3 signal pathway in lung cancer cells was involved in the protection of macrophages.

### IL-10-STAT1/STAT3 axis protected tumor cells *via* alleviating ER stress induced by CAP

To further explore the mechanism of macrophages protecting tumor cells from CAP, the co-cultured and separately cultured Calu-1 cells were collected at 6 h after CAP (30 s) treatment to perform RNA-seq analysis.

**Fig. 4:**
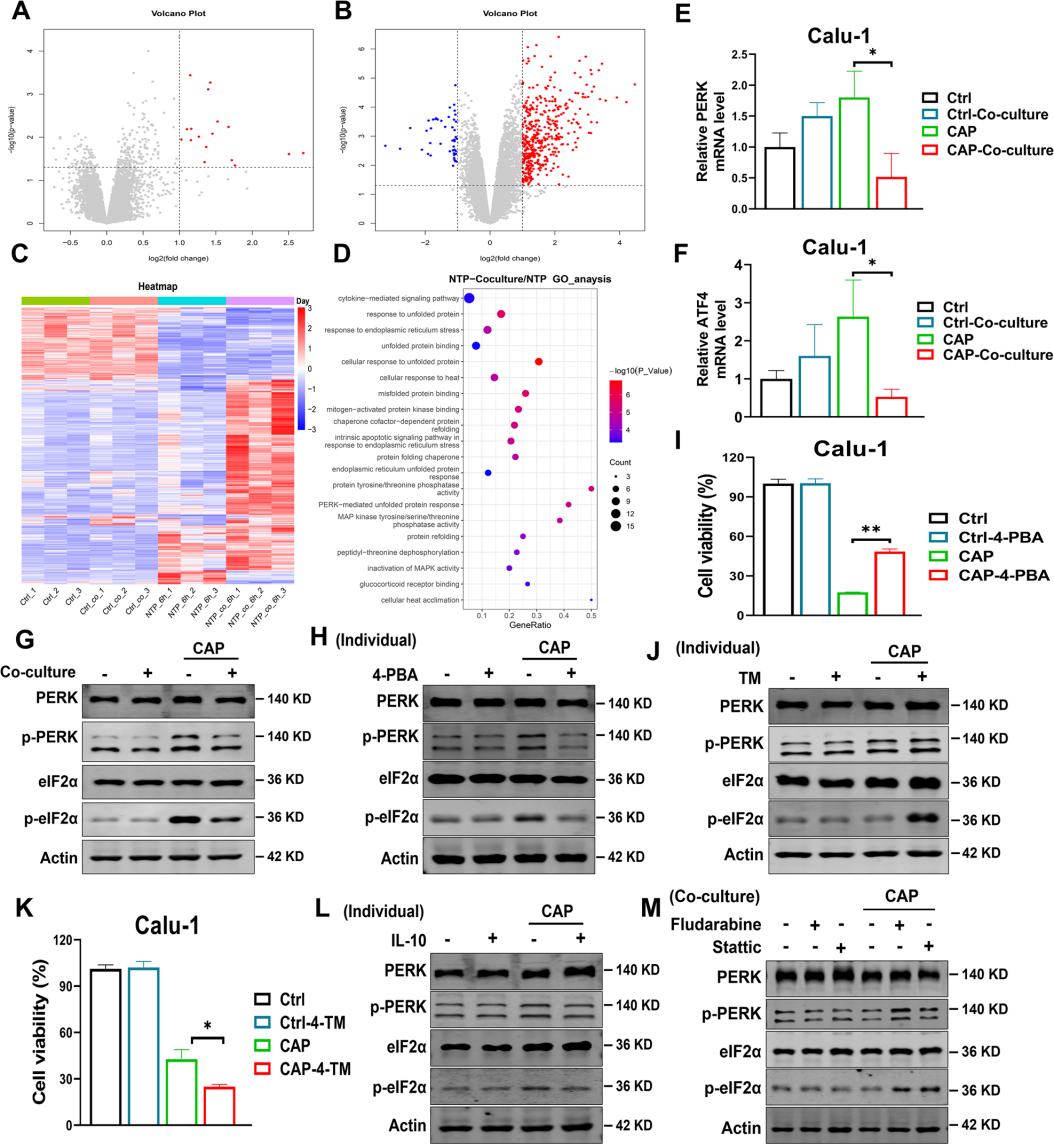
IL-10-STAT1/STAT3 axis protects tumor cells *via* alleviating ER stress induced by CAP. **A, B** The differentially expressed genes enriched in the separately cultured (**A**) and co-cultured (**B**) Calu-1 cells after CAP treatment. **C** Heat map of gene expressions in each treatment group. **D** ER stress-related pathways enriched by the GO enrichment analysis. **E, F** Transcription level of PERK (**E**) and ATF4 (**F**). **G** Protein expression of PERK, p-PERK, eIF2α and p-eIF2α. **H, I** Effect of 4-PBA (4 μM) on protein expression of PERK, p-PERK, eIF2α and p-eIF2α (**H**) and the viability (**I**) of separately cultured Calu-1 cells after CAP treatment. **J, K** Effect of TM (3 μM) on protein expression of PERK, p-PERK, eIF2α and p-eIF2α (**J**) and the viability (**K**) of co-cultured Calu-1 cells after CAP treatment. **L** Effect of IL-10 (20 μg/mL), on protein expression of PERK, p-PERK, eIF2α and p-eIF2α in separately cultured Calu-1cells after CAP treatment. **M** Effect of Fludarabine (10 mM) or Stattic (30 mM) on protein expression of PERK, p-PERK, eIF2α and p-eIF2α in co-cultured Calu-1cells after CAP treatment. *: *p* < 0.05; **: *p* < 0.01.

The results of DEGs analysis showed significant alterations in gene expression profiles, with 1388 genes significantly upregulated in the co-culture group (log FC > 0.8, *p* < 0.05) while 228 genes were significantly downregulated (Fig. 4A-C) after CAP treatment compared to the separately cultured Calu-1 cells. The regulation of cytokine receptor action, endoplasmic reticulum (ER) protein synthesis, PERK mediated ER stress response, and unfolded protein response (UPR) were all significantly enriched by GO enrichment analysis (Fig. 4D) in the co-culture group after CAP treatment.

Furthermore, the expression levels of ER stress-related genes were analyzed and the results showed that the transcription levels of PERK and ATF4 in the co-cultured Calu-1 cells were significantly decreased at 6 h after CAP treatment, compared to those of separately cultured Calu-1 cells (Fig. 4E, F). Meanwhile, the phosphorylation levels of ER stress-related proteins, including PERK and eIF2α, in the co-culture group were also significantly reduced after CAP treatment (Fig. 4G).

Based on these results, we supposed that macrophages might protect the co-cultured tumor cells *via* alleviating the ER stress induced by CAP. To verify it, pretreatment of separately cultured Calu-1 cells with the ER stress inhibitor 4-phenylbutyric acid (4-PBA, 5 μM) for 12 h prior to CAP exposure, and the cell viability and the expression of ER stress related proteins were detected at 24 h after CAP treatment. As showed in Fig. 4H, I, 4-PBA treatment significantly reduced the phosphorylation levels of PERK and eIF2α (Fig. 4H) in the CAP-treated cells (separately cultured), and the viability of cells with 4-PBA treatment was distinctly higher than that without 4-PBA treatment after CAP treatment (Fig. 4I). On the contrary, treatment with an ER stress agonist, Tunicamycin (TM, 3 μM), elevated the phosphorylation levels of PERK and eIF2α (Fig. 4J) and decreased the viability of Calu-1 cells co-cultured with macrophages after CAP treatment (Fig. 4K). These results indicate that macrophages might protect the co-cultured tumor cells *via* alleviating ER stress, which might mediate the killing effect of CAP.

Previous studies showed that IL-10 could attenuate ER stress and suppress apoptosis induced by Doxorubicin treatment *via* STAT1/STAT3 signaling pathway [22]. To determine the role of IL-10-STAT1/STAT3 axis in ER stress induced by CAP treatment in tumor cells, we treated the separately cultured Calu-1 cells with exogenous IL-10 (20 μg/mL) to mimic the possible release of IL-10 from CAP-treated macrophage. Results showed that IL-10 treatment reduced the phosphorylation levels of PERK and eIF2α proteins detected at 6 h after CAP treatment (Fig. 4L). However, treatment with Fludarabine (10 μM) or Stattic (30 μM) for Calu-1 cells before co-culture elevated the phosphorylation levels of these proteins after CAP treatment (Fig. 4M). These results indicate that macrophage-derived IL-10 alleviates CAP-induced ER stress in tumor cells through activation of the STAT1/STAT3 signaling axis.

### Macrophages suppressed CAP-induced apoptosis, pyroptosis and autophagy in tumor cells *via* alleviating ER stress

**Fig. 5:**
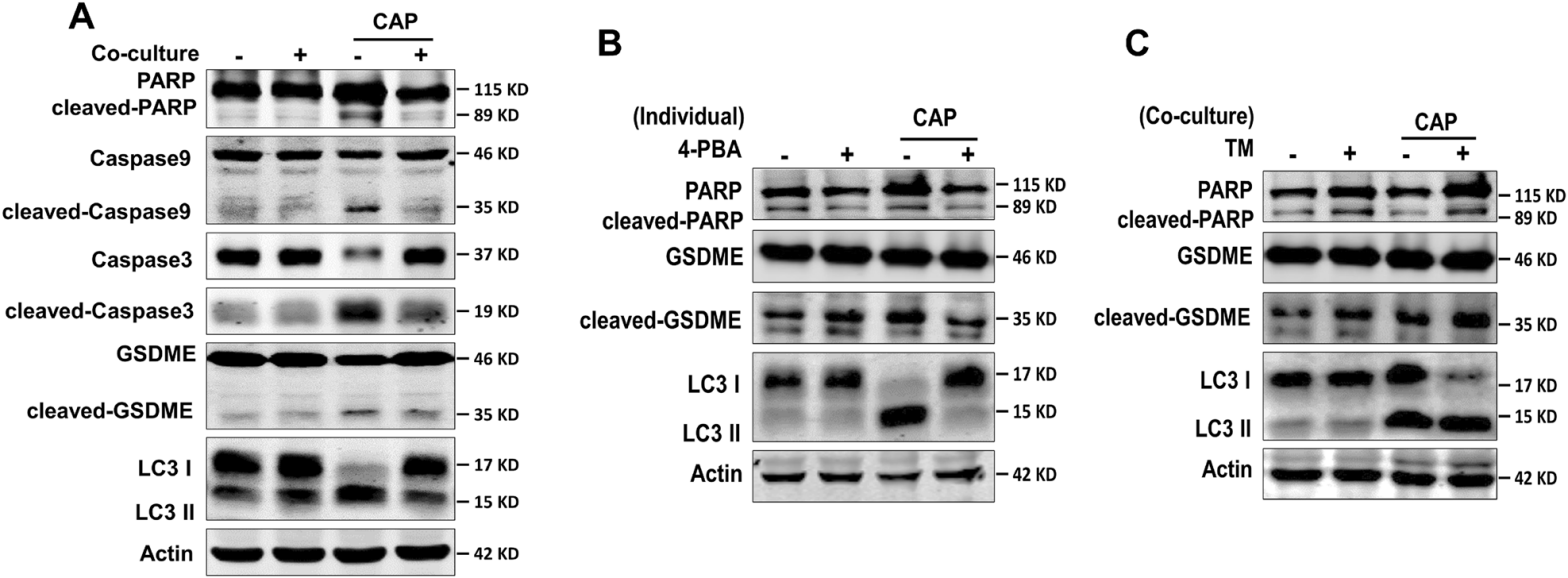
Macrophages inhibit CAP-induced apoptosis, pyroptosis and autophagy in tumor cells *via* alleviating ER stress. **A** Protein expression of apoptosis, pyroptosis and autophagy in Calu-1 cells after CAP treatment. **B** Effect of 4-PBA (4 μM) on protein expression of apoptosis, pyroptosis and autophagy in the separately cultured Calu-1 cells after CAP treatment. **C** Effect of TM (3 μM) on protein expression of apoptosis, pyroptosis and autophagy in the co-cultured Calu-1 cells after CAP treatment.

Considering that CAP treatment could induce various types of programmed cell death, including apoptosis, ferroptosis and pyroptosis, etc. [1], it was pertinent to further explore which cell death types were regulated by the co-cultured macrophages after CAP treatment. We detected the expression of marker proteins of various types of cell death. The results revealed that macrophages significantly attenuated CAP-induced activation of multiple cell death pathways in Calu-1 cells. Specifically, the protein expression levels which were related to apoptosis (cleaved-Caspase 9, cleaved-Caspase 3 and cleaved-PARP), pyroptosis (cleaved-Caspase 3 and cleaved-GSDME) and autophagy (LC3-II/I) in the co-culture Calu-1 cells were evidently lower than those of separately cultured Calu-1 cells after CAP treatment (Fig. 5A).

To determine whether alleviation of ER stress mediated the decrease of apoptosis, pyroptosis and autophagy after CAP treatment, the separately cultured Calu-1 cells were treated with 4-PBA. The results in Fig. 5B showed that 4-PBA markedly attenuated CAP-triggered activation of apoptosis (cleaved-PARP), pyroptosis (cleaved-GSDME), and autophagy (LC3-II/I) pathways. Conversely, TM pretreatment upregulated the expression of apoptosis, pyroptosis and autophagy proteins in the co-cultured Calu-1 cells after CAP treatment (Fig. 5C). These results indicate that CAP-induced ER stress plays an important role in the occurrence of these types of cell death.

### Macrophages attenuated the tumor suppression effect of CAP in vivo

**Fig. 6:**
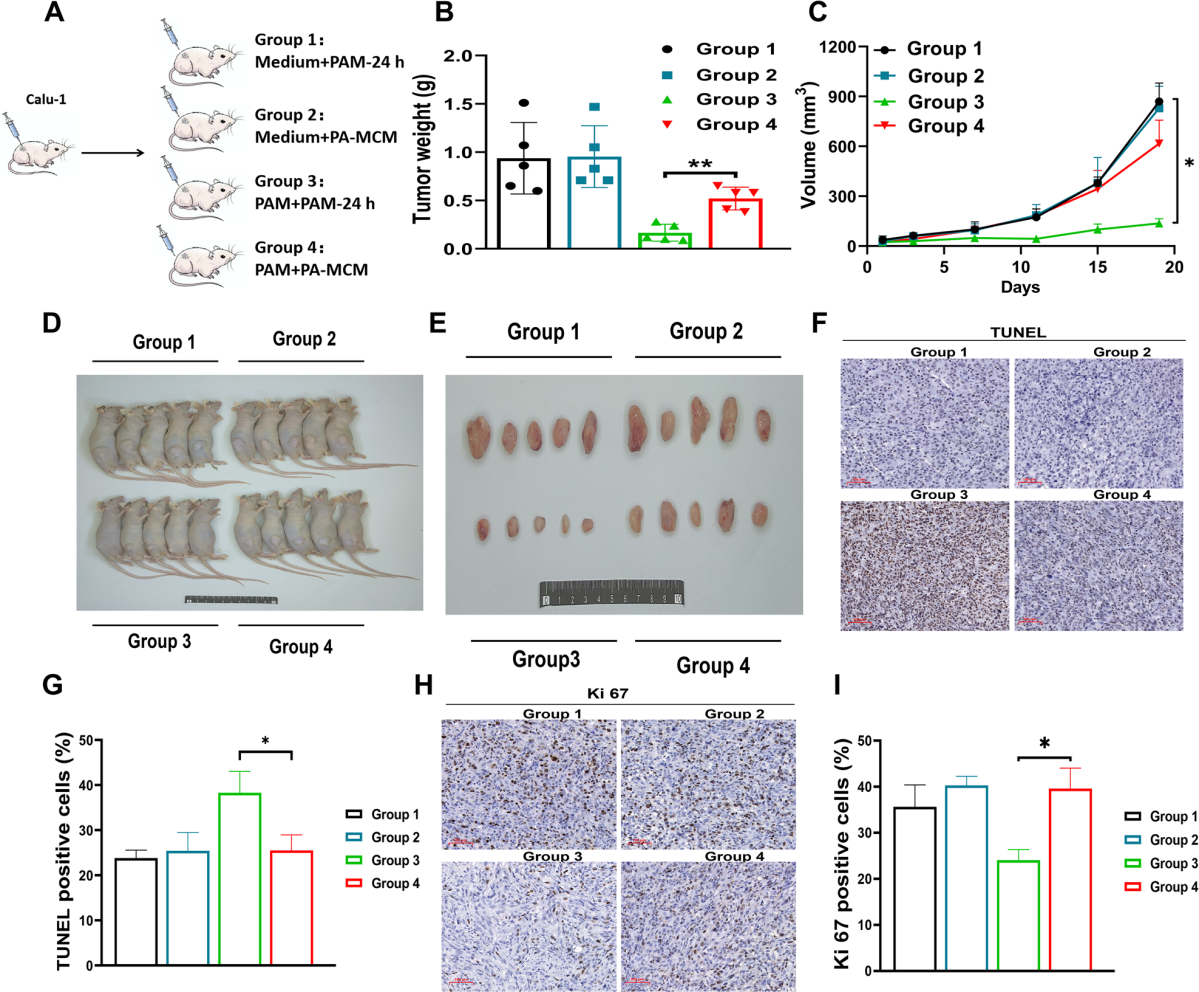
Macrophages attenuate the tumor suppression effect of CAP in tumor-bearing mice. **A** Scheme of the group setting. PAM: the RPMI-1640 medium treated by CAP for 30 s; MCM: the M0 macrophages culture medium collected after 24 h incubation; PAM-24 h: the plasma-activated RPMI-1640 medium placed in the cell incubator for 24 h was labeled as PAM-24 h to distinguish from the freshly made PAM; PA-MCM: the M0 macrophages incubated with fresh PAM (10 mL) for 24 h, and the supernatant was collected and labelled as PA-MCM. **B** Tumor growth curve, *n* = 5. **C** The weight of excised tumors. **D** The images of nude mice. **E** The images of excised tumors. **F** Representative images of TUNEL and the quantified results (**G**), Scale bar: 100 μm. **H** Representative IHC images of Ki 67 and the quantified results (**I**), Scale bar, 100 μm. ns no significance *: *p* < 0.05; **: *p* < 0.01.

To further explore the effect of macrophages in vivo, we established the tumor xenograft model (Calu-1) in nude mice and treated the xenografts with the corresponding activated medium (once every day) for 19 days (Fig. 6A). Comparative analysis revealed that average tumor volume in PA-MCM treatment group was significantly higher than that of the PAM treatment group (Fig. 6B-E), while the weight of mice almost showed little difference (Extended Fig. 6), indicating that macrophages could effectively attenuate the tumor suppression effect of CAP treatment in vivo. Furthermore, the fraction of TUNEL positive cells in the PAM treatment group was much higher than that in the PA-MCM treatment group (Fig. 6F, G), while the fraction of Ki67 positive cells was significantly lower, indicating that the presence of macrophages reduces the tumor cell death and the suppressive effect on tumor cell proliferation induced by CAP treatment in vivo (Fig. 6H, I).

## Discussion

As a new potential technology for tumor treatment, CAP, which could introduce ROS/RNS into tumor cells to induce damage and dysfunction (including mitochondria, endoplasmic reticulum and lysosome) to organelles and finally lead to various programmed death of tumor cells, had been developed rapidly [23–26]. Tornin et al. proved that CAP inhibited cell growth and induced apoptosis *via* activating AMPK or STAT3 signaling pathway in osteosarcoma cells [8]. Adhikari et al. reported that the synergistic effect of CAP and silymarin nanoemulsion could induce autophagy through activating the PI3K and EGFR signaling pathways in G-361 cells [27]. Previously, our group revealed that CAP caused GSDME-dependent pyroptosis through ROS/caspase-9/caspase-3/GSDME signaling pathway for the first time [28]. Recently, we also discovered that combinatorial treatment with CAP and low-dose RSL3 could provoke ferroptosis *via* promoting xCT lysosomal degradation through ROS/AMPK/mTOR axis in lung cancer cells [1]. Furthermore, mitotic catastrophe induced by low dose CAP treatment was firstly discovered by us to effectively suppress the tumor growth [29]. In contrast one type of cell death being reported in these previous studies, the results of our current study confirmed that CAP treatment could induce multiple types of cell death simultaneously, including apoptosis, autophagy and pyroptosis (Fig. 5A), indicating the complexity of CAP-induced programmed cells death.

In addition, damages of organelles caused by CAP are key causes for the death of tumor cells. Patrakova et al. reported the synergism of CAP and chloroquine could promote the death of A549 cells *via* inhibiting the expression of mitochondrial protein and resulting in mitochondrial dysfunction [30]. Kim et al. proved that CAP caused death of myeloid leukemia cells through regulating the protein expression of RING126 and the dysfunction of lysosome [31]. Currently, some studies showed that CAP treatment induced cell death mediated by organelle dysfunction and ER stress through upregulation of unfolded protein response (UPR) levels, promotion of ROS and Ca^2+^ accumulation, and reduction of intracellular pH in tumor cells [32, 33]. Interestingly, our study revealed a novel finding that CAP also induced distinctly increased ER stress and multiple programmed cell deaths in lung cancer cells, while the agonists or inhibitors of ER stress could significantly enhance or attenuate the levels of apoptosis, autophagy, and pyroptosis (Fig. 5). Our results suggested that ER stress should play as a central regulator orchestrating CAP-induced programmed cell death in lung cancer cells.

Currently, the polarization of macrophages was found to be important in tumor treatment [34]. In TME, M2 macrophage facilitated the development and malignancy of tumor cell *via* releasing IL-10, TGF-β and other anti-inflammatory cytokines, while M1 polarization of macrophage was induced by some clinical chemotherapy drugs to exhibit the anti-tumor effect [35]. Wang et al. reported that vinblastine could reset TAM toward M1 phenotype and promote anti-tumor immune response *via* producing ROS and activating NF-κB signaling pathway [36]. It is a crucial and efficacious strategy to exhibit anti-tumor effect by modulating the macrophage polarization.

As the largest proportion of immune cells in TME, macrophages will inevitably be exposed to CAP in the course of tumor treatment. As we know, the polarization of macrophages displays phenotypic plasticity, with its states existing in a dynamic equilibrium in response to TME stimuli [37]. Exposure to different cytokines or the change of TME would lead to transformation of the macrophage polarization states [38]. Meanwhile, the effects of CAP treatment on macrophage polarization are also not unchanging but variable depending on the parameter settings of the experiment, which include the type of plasma devices (jet plasma, DBD plasma, etc.), the treatment manners (direct treatment with plasma, or indirect treatment with plasma-activated solution), dose parameters (high dose or low dose), timepoints for experimental detection, and so on. [39].

**Fig. 7:**
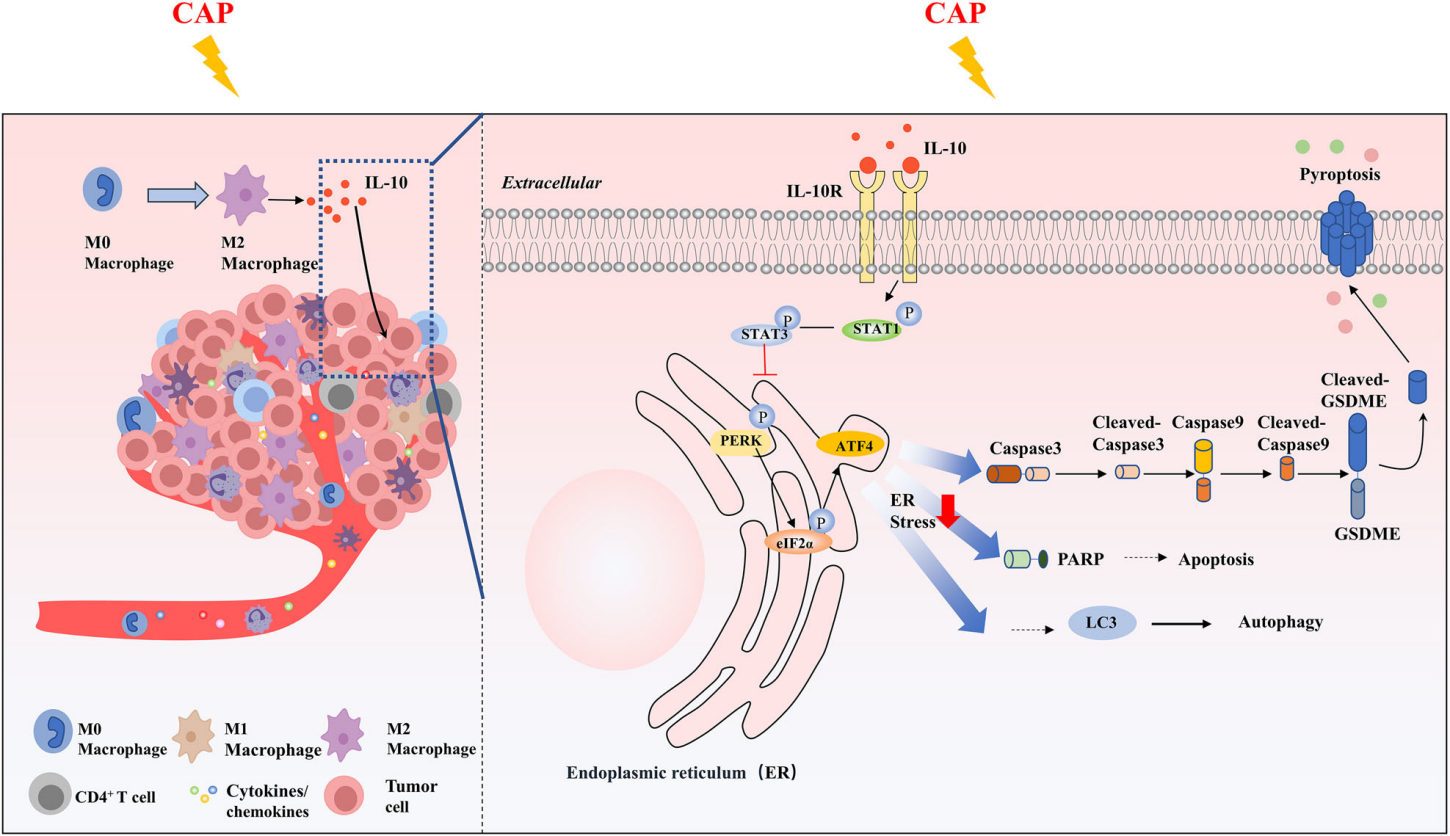
Macrophages attenuate the anti-tumor effect of CAP treatment.

Hence, exploring the roles of macrophage in CAP anti-tumor therapy and the possible underlying mechanisms will be helpful for the understanding of the anti-tumor mechanism of CAP in the complicated TME in vivo and for promoting clinical applications of CAP treatment [40]. Modulation of macrophage polarization is still not well understood currently. Kaushik et al. found that CAP induced M1 polarization of macrophage and effectively enhanced the death of glioma cells *via* secreting TNF-α [41, 42]. However, Crestale et al. reported that CAP could induce polarization of macrophage into the anti-inflammatory M2 phenotype [19]. In this study, we first systematically investigated the effect of macrophages on CAP treatment in the co-culture system of macrophages and tumor cells. We found that macrophages significantly alleviated the killing effect of CAP on tumor cells (Fig. 1). In addition, we also revealed that this phenomenon was mediated by IL-10 from the M2 polarization of macrophages induced by CAP treatment. The further study found that the STAT1/STAT3 signaling pathway in tumor cells activated by IL-10 inhibited the PERK signaling pathway and alleviated the ER stress-mediated cell death in tumor cells caused by CAP treatment (Fig. 7). These results provide novel insights into the complicated interaction between CAP and macrophages within the TME.

These studies revealed an important therapeutic challenge. Induction of M2 macrophages might raise some new questions and challenges to the clinical application of CAP tumor treatment, and also suggest that the combination of chemotherapy drugs regulating M1 polarization of macrophages might be a promising strategy to improve the therapeutic effect of CAP in clinical applications.

## Conclusions

Our research revealed a regulatory cascade that has not been fully recognized during CAP treatment. Specifically, the macrophages were induced to M2 polarization after CAP treatment. After that, IL-10 (an effective anti-inflammatory cytokine) released by these M2 macrophages was recognized and caught by its specific receptors on the membrane of tumor cells, thereby activating the STAT1/STAT3 signaling pathway, which then alleviated the level of ER stress in tumor cells induced by CAP treatment, restored cellular homeostasis and rescued the tumor cells from multiple programmed cell death modes.

In summary, our study revealed a significant mechanism in CAP treatment. It not only broadens and deepens the understanding on the complicated biological effects of CAP on polarization and activation of macrophages, but also unveils a novel mechanism by which immune cells influence on the anti-tumor efficacy of CAP treatment. These findings raise a potential problem during CAP treatment, promote our further perception on CAP, and provide some theoretical foundation for developing innovative strategies for CAP clinical application.

## Materials and methods

### Cell culture

THP-1 cell line was purchased from the Cell Bank of Type Culture Collection of the Chinese Academy of Sciences (Shanghai, China). Calu-1, H1299 and H1975 cell lines were obtained from the American Type Culture Collection (Manassas, USA). All these cell lines were cultured in RPMI-1640 medium, supplemented with 100 μ/mL penicillin and 100 μg/mL streptomycin with 10% FBS, and were maintained at 37 °C in 5% CO_2_.

### CAP equipment and treatment

The atmospheric pressure dielectric barrier discharge (DBD) device used in this study mainly consisted of three parts, including the reaction chamber, high voltage power supply and the gas source. The high voltage electrode of the reaction chamber was 32-mm diameter copper column and CAP was generated by the voltage of 12 kV (peak to peak) with the frequency of 24 kHz. The discharge gap between the bottom of quartz glass and the surface of the medium was maintained at 5 mm. The high-purity helium (99.999%) was used as working gas with a gas flow rate of 120 L/h. The complete medium (1.5 mL) was treated with CAP for 30 s to prepare plasma-activated medium (PAM).

### Co-culture system of macrophages and tumor cells

As shown in Fig. 1A, the co-culture system with transwell insert (0.4 μm pore size; Corning, USA) was established. THP-1 cells (1 × 10^6^) were seeded firstly into insert and differentiated into M0 macrophages after PMA (100 ng/mL) treatment for 24 h. The tumor cells (3 × 10^6^) were seeded into the wells of six-well plate and co-cultured with the inserts with THP-1-derived macrophages in, and then the freshly made plasma-activated medium (PAM, 3.5 mL) was added into the co-culture system.

### Statistical analysis

All experiments were performed at least three times, and data was analyzed with GraphPad Prism 8 statistical software (San Diego, CA, USA). The significance of data in each group was analyzed with *t*-test and a *p* value less than 0.05 was considered as significant difference. Data were expressed as the means ± SD of triplicates.

## Supplementary information

Extended Table-1

Supplementary information

Extended Table-2

Extended figure 1

Extended figure 2

Extended figure 3

Extended figure 4

Extended figure 5

Extended figure 6

WB Original data

## Data Availability

Data will be made available on request. The raw sequence data reported in this paper have been deposited in the Genome Sequence Archive (Genomics, Proteomics & Bioinformatics 2021) in National Genomics Data Center (Nucleic Acids Res 2025), China National Center for Bioinformation / Beijing Institute of Genomics, Chinese Academy of Sciences (GSA-Human: HRA012578) that are publicly accessible at https://ngdc.cncb.ac.cn/gsa-human.
