# Supplementary material for: M2 polarization of macrophage protects the lung cancer cells from cold atmospheric plasma via alleviating endoplasmic reticulum stress
Source: Cell Death Discov. 2025 Oct 27;11:487. doi: 10.1038/s41420-025-02775-4 (PMC12559389; doi:10.1038/s41420-025-02775-4)
Supplement: Supplementary file 1 — Extended Table-1 [file 41420_2025_2775_MOESM1_ESM.docx]

Extended Table 1

| Primer | Sequence |
| --- | --- |
| IL-10 | Forward Primer:5’-TCAAGGCGCATGTGAACTCC-3’  Reverse Primer: 5’-GATGTCAAACTCACTCATGGC-3’ |
| IL-10R1 | Forward Primer: 5’-CCTCCGTCTGTGTGGTTTGAA-3’  Reverse Primer:5’-CACTGCGGTAAGGTCATAGGA-3’ |
| TGF-β1 | Forward Primer: 5’-CTAATGGTGGAAACCCACAA-3’  Reverse Primer: 5’-TATCGCCAGGAATTGTTGCTG-3’ |
| CCL1 | Forward Primer: 5’-ACCAGCTCCATCTGCTCCAAT-3’  Reverse Primer: 5’-TGTGCCTCTGAACCCATCCA-3’ |
| CCL18 | Forward Primer: 5’-CTATACCTCCTGGCAGATTC-3’  Reverse Primer: 5’-CTCTCTTGGTTAGGAGGATG-3’ |
| CXCL13 | Forward Primer: 5’-GAGGCAGATGGAACTTGAGC-3’  Reverse Primer: 5’-CTGGGGATCTTCGAATGCTA-3’ |
| CCL17 | Forward Primer: 5’-TTCTCTGCAGCACATCCACG-3’  Reverse Primer: 5’-CTGGAGCAGTCCTCAGATGT-3’ |
| CCL22 | Forward Primer:5’-TCCTGGGTTCAAGCGATTCTC-3’  Reverse Primer: 5’-GTCAGGAGTTCAAGACCAGC-3’ |
| CCL24 | Forward Primer:5’-GGACTCTTATTGGCCGCCTTCC-3’  Reverse Primer: 5’-CGGGCATGGTGACTGGGATTT-3’ |
| PERK | Forward Primer: 5’-ACGATGAGACAGAGTTGCGA-3’  Reverse Primer: 5’-ATCCAAGGCAGCAATTCTCCC-3’ |
| ATF4 | Forward Primer: 5’-TTCTCCAGCGACAAGGCTAA-3’  Reverse Primer: 5’-CTCCAACATCCAATCTGTCCC-3’ |
| GAPDH | Forward Primer: 5’-ACAACTTTGGTATCGTGGA-3’  Reverse Primer: 5’-GCCATCACGCCACAGTTTC-3’ |
