# Supplementary material for: M2 polarization of macrophage protects the lung cancer cells from cold atmospheric plasma via alleviating endoplasmic reticulum stress
Source: Cell Death Discov. 2025 Oct 27;11:487. doi: 10.1038/s41420-025-02775-4 (PMC12559389; doi:10.1038/s41420-025-02775-4)
Supplement: Supplementary file 2 — Supplementary information [file 41420_2025_2775_MOESM2_ESM.doc]

**Supplementary Information**

1. **Abbreviation**

CAP Cold atmospheric plasma

PAM Plasma-activated medium

PMA Phorbol 12-Myristate 13-Acetate

LPS Lipopolysaccharide

ER stress Endoplasmic reticulum stress

GO enrichment Gene Ontology enrichment

1. **Materials and Methods**

**2.1 Reagents and antibodies**

Lipopolysaccharides (LPS, HY-D1056), Phorbol 12-myristate 13-acetate (PMA, HY-18739), IL-10 (HY-P70751), TGF-β1 (HY-P7118), 4-Phenylbutyric acid (HY-A0281), Tunicamycin (HY-A0098), Fludarabin (HY-B0069) and Stattic (HY-13818) were all obtained from MedChemExpress (New Jersey, USA). IFN-γ (DC014), IL-4 (CX03) and IL-13 (CC89) were purchased from Novoprotein (Suzhou, China). Human Fc block (564219) was obtained from Bioscience (New Jersey, USA). Pr9opidium Iodide (PI, A601112) was purchased from Sangon Biotech (Shanghai, China). TUNEL test kit was obtained from Keygen Biotech (KGA703, Jiangsu, China). ELISA test kit was purchased from Elabscience (E-EL-H6154).

The primary antibodies, anti-PARP (9532), anti-Caspase 3 (9662), anti-Caspase 9 (9502), anti-STAT1 (9167), anti-p-STAT1 (9176), anti-STAT3 (9139) and anti-p-STAT3 (9145) were all purchased from Cell Signaling Technology (CST, Beverly, USA). The anti-GSDME antibody (ab215191) was obtained from Abcam (Cambridge, USA). The anti-β-actin (HA601082) was bought from HuaBio (Hangzhou, China). The anti-p-JAK1 (E-AB-20913) was obtained from Elabscience (Wuhan, China). The anti-JAK1 (66466-1-Ig), anti-PERK (24390-1-AP), anti-eIF2α (11170-1-AP), anti-p-PERK (29546-1-AP), anti-p-eIF2α (28740-1-AP), anti-IL-10R1 (13356-1-AP) and anti-LC3 (14600-1-AP) were obtained from Proteintech (Wuhan, China). The anti-rabbit (926-32211) and anti-mouse (926-68070) secondary antibodies were acquired from Li-COR (Lincoln, NE, USA). The anti-PE-CD206 (12-2069-42) and anti-FITC-CD80 (11-0809-42) were obtained from eBioscience (Waltham, MA, USA). The Ki 67 antibody (MAB-0672) was purchased from Biotechnologies (Fuzhou, China).

**2.2 Cell viability detection**

Cell viability was detected with a Cell Counting Kit-8 (CCK-8) kit (TargetMoi, USA) following the manufacturer’s instructions. The cells were incubated with CCK-8 solution (1 mg/mL) at 37℃ for 25 min and then the absorbance at 450 nm was measured with a Varioskan Flash microplate reader (Thermo Fisher Scientific, Rockford, IL, USA).

**2.3 Cell death detection**

Cells were collected, washed with PBS and stained with PI solution (5 μg/mL) for 20 min at room temperature, and assessed with flow cytometry (Accuri C6, BD Biosciences, Bedford, MA, USA) according to the manufacturer’s instructions. Data were processed with FlowJo software (TreeStar, Ashland, OR, USA).

**2.4 Macrophage polarization and detection**

M0 Macrophages were treated with IFN-γ (20 ng/mL) + LPS (10 ng/mL) or IL-4 (20 ng/mL) + IL-13 (20 ng/mL) to induce M1 or M2 macrophages as positive controls, respectively ^[20]^. After PAM treatment, macrophages (1×10^6^) were collected, washed with PBS and then blocked with human Fc block buffer (Biosciences, USA) at 4℃ for 15 min. Subsequently, cells were incubated with FITC-CD80 and PE-CD206 antibodies (eBioscience, USA) at 4℃ for 30 min, and then analyzed with flow cytometry.

**2.5 Quantitative real-time PCR analysis (qRT-PCR)**

Total RNA was extracted with a RNeasy Mini kit (Qiagen, Germany) following the manufacturer’s instructions, and cDNA was synthesized with ProtoScript First Strand cDNA Synthesis Kit (Yeasen, China). The reaction mixtures of qRT-PCR were prepared with Hieff qPCR SYBR Green Master Mix (Yeasen, China). The reaction was performed and analyzed with Light Cycler 480 Instrument (Roche, Basel, Switzerland). The GADPH gene was set as control, and the relative mRNA levels were normalized to GADPH mRNA level. The fold changes of mRNA were calculated with the 2^-ΔΔCt^ method. All primers used in this study are listed in Extended Table 1.

**2.6 Western blot**

Cells were collected and lysed with RIPA lysis buffer within PMSF (1 mmol/L). The lysates were centrifugated at 14500 rpm at 4℃ for 10 min and the supernatants were collected. The protein concentration was measured with a BCA Protein Assay Reagent Kit (Beyotime Biotechnology, China). The protein extractions (40 μg) were separated with SDS-PAGE (Beyotime, USA), and blocked with 5% nonfat milk at room temperature for 1 h, then incubated with antibodies, respectively. The protein bands were visualized with an Odyssey® CLx Infrared Imaging System (Li-COR, Lincoln, USA).

**2.7 Small interfering RNA transfection**

The siRNA for IL-10R1 and the NC siRNA were synthesized by Suzhou Jima Genomics Company (China). The siRNA sequence of IL-10R1 was as follows: 5'-CCAGGCAGCTGATCATAGA-3'. Tumor cells were transfected with IL-10R1 siRNAs with Lipofectamine 2000 transfection reagent (Thermo, USA) according to the manufacturer’s protocol.

**2.8** **Animal experiment**

Calu-1 cells (1×10^7^ cells), suspended in 100 µL PBS, were injected into the right hind flank of BALB/c nude mice (male, 5-6 weeks of age) obtained from GemPharmatech Company (Nanjing, China). Mice were randomly divided into four groups. Group 1: RPMI-1640 medium (100 μL) + PAM-24 h (plasma-activated RPMI-1640 medium after laying aside in the cell incubator for 24 h, 100 μL). Group 2: RPMI-1640 medium (100 μL) + PA-MCM (plasma-activated macrophage conditioned RPMI-1640 medium, 100 μL). Group 3: fresh PAM (plasma-activated RPMI-1640 medium, 100 μL) + PAM-24 h (100 μL). Group 4: fresh PAM (100 μL) + PA-MCM (100 μL). There are 5 mice in each group, and the mice were daily injected with the corresponding liquid (totally 200 μL) for 19 days. Subsequently, all mice were sacrificed and the subcutaneous transplanted tumors were isolated for the next experiments. The preparation of all injected fluids used in the animal experiment were described in Extended Table 1. All animal experiments have been approval by Hefei Institutes of Physical Science Experimental Animal Ethics Committee.

Extended Table 1

| Liquid type | Preparation method |
| --- | --- |
| PAM | the RPMI-1640 medium treated by CAP for 30 s. |
| MCM | the M0 macrophages culture medium collected after 24 h incubation. |
| PAM-24 h | the plasma-activated RPMI-1640 medium placed in the cell incubator for 24 h, labeled as PAM-24 h to distinguish from the freshly made PAM. |
| PA-MCM | the M0 macrophages incubated with fresh PAM (10 mL) for 24 h, and the supernatant was collected and labelled as PA-MCM. |

**2.9 Immunohistochemistry**

Tumor sections (5 mm) were deparaffinized in xylene, rehydrated by gradient ethanol, antigen repaired through citrate solution, blocked with 3% hydrogen peroxide solution, and then stained with Ki 67 antibody (Keygen Biotech, Nanjing, China) or TUNEL kit (Key Gene Bio Tech, Fuzhou, China) according to manufacturer’s instructions, respectively.

**2.10 Primers for PCR amplification**

The primers for PCR amplification are shown as Extended Table 2.

**Extended Table 2**

| Primer | Sequence |
| --- | --- |
| IL-10 | Forward Primer:5’-TCAAGGCGCATGTGAACTCC-3’  Reverse Primer: 5’-GATGTCAAACTCACTCATGGC-3’ |
| IL-10R1 | Forward Primer: 5’-CCTCCGTCTGTGTGGTTTGAA-3’  Reverse Primer:5’-CACTGCGGTAAGGTCATAGGA-3’ |
| TGF-β1 | Forward Primer: 5’-CTAATGGTGGAAACCCACAA-3’  Reverse Primer: 5’-TATCGCCAGGAATTGTTGCTG-3’ |
| CCL1 | Forward Primer: 5’-ACCAGCTCCATCTGCTCCAAT-3’  Reverse Primer: 5’-TGTGCCTCTGAACCCATCCA-3’ |
| CCL18 | Forward Primer: 5’-CTATACCTCCTGGCAGATTC-3’  Reverse Primer: 5’-CTCTCTTGGTTAGGAGGATG-3’ |
| CXCL13 | Forward Primer: 5’-GAGGCAGATGGAACTTGAGC-3’  Reverse Primer: 5’-CTGGGGATCTTCGAATGCTA-3’ |
| CCL17 | Forward Primer: 5’-TTCTCTGCAGCACATCCACG-3’  Reverse Primer: 5’-CTGGAGCAGTCCTCAGATGT-3’ |
| CCL22 | Forward Primer:5’-TCCTGGGTTCAAGCGATTCTC-3’  Reverse Primer: 5’-GTCAGGAGTTCAAGACCAGC-3’ |
| CCL24 | Forward Primer:5’-GGACTCTTATTGGCCGCCTTCC-3’  Reverse Primer: 5’-CGGGCATGGTGACTGGGATTT-3’ |
| PERK | Forward Primer: 5’-ACGATGAGACAGAGTTGCGA-3’  Reverse Primer: 5’-ATCCAAGGCAGCAATTCTCCC-3’ |
| ATF4 | Forward Primer: 5’-TTCTCCAGCGACAAGGCTAA-3’  Reverse Primer: 5’-CTCCAACATCCAATCTGTCCC-3’ |
| GAPDH | Forward Primer: 5’-ACAACTTTGGTATCGTGGA-3’  Reverse Primer: 5’-GCCATCACGCCACAGTTTC-3’ |

**3. Results**

**3.1 Macrophages significantly reduced the killing effect of CAP on tumor cells**

Separately cultured and co-cultured tumor cells (H1299 and H1975) were assessed with CCK-8 and PI staining at 24 h after CAP treatment. Results in Extended Figure 1 showed that CAP treatment (30 s) significantly reduced the viability of H1299 cells into 20.9 ± 3.6% of control while inducing 57.9 ± 5.9% PI positive cells (Extended Figure 1A&B). Similarly, CAP treatment (30 s) decreased the viability of H1975 cells to 17.1 ± 4.2% of control and induced 47.1 ± 2.4% PI positive cells (dead cells) (Extended Figure 1C&D).

However, the presence of macrophages in the co-culture system significantly attenuated the viability decrease of H1299 and H1975 cells after CAP exposure (Extended Figure 1A&B). This protective effect was further confirmed by PI staining, which revealed the distinctly decreased cell death of H1299 cells co-cultured with macrophages compared to that of separately cultured H1299 and H1975 cells after CAP treatment (Extended Figure 1 C&D).

**3.2 The induction of macrophages polarization**

CAP treatment (30 s) induced distinct morphological changes in macrophages, causing an elongated, oval or spindle-shaped appearance (Extended Figure 2A). To evaluate how M2 polarization affects co-cultured Calu-1 cells, we first polarized M0 macrophages to either M1 or M2 type by treating with LPS + IFN-γ or IL-4 + IL-13 for 48 h (Extended Figure 2B~G), respectively, and then co-cultured with Calu-1 cells for CAP treatment (30 s).

The results demonstrated that CD80 rather than CD206 positive cells increased distinctly after LPS + IFN-γ treatment for 48 h (Extended Figure 2B~D), indicating that the polarization of M1 type was induced successfully. Meanwhile, CD206 but not CD80 positive cells increased distinctly after IL-4 + IL-13 treatment for 48 h (Extended Figure 2E~G). These distinct marker profiles confirmed the effective generation of M1 and M2 macrophage populations, respectively.

**3.3** **Macrophages attenuate the killing effect of CAP through IL-10-STAT1/STAT3 pathway**

To better characterize the secretion pattern of cytokines, we quantified the expression level of IL-10 in THP-1 cell culture supernatants by ELISA. The result revealed that CAP treatment significantly promoted IL-10 secretion in THP-1 cells, a similar trend was observed in the co-culture system (Extended Figure 3A). This demonstrated that CAP treatment induced M2 polarization of THP-1 cells and the subsequent secretion of IL-10.

**3.4 CAP induced autophagic cell death in tumor cells after CAP treatment**

In order to explore the type of autophagy induced by CAP, the separately cultured Calu-1 cells were pretreated with NH_4_Cl (an autophagy inhibitor, 100 mM) for 4 h and the viability of Calu-1 cells was detected after CAP treatment for 20 h. The results in Extended Figure 4 showed that the viability of Calu-1 cells with NH_4_Cl-pretreated was distinctly higher than that without NH_4_Cl treatment after CAP treatment. The results indicate that CAP induced autophagic cell death in Calu-1 cells.

**3.5 IL-10-STAT1/STAT3 pathway inhibits CAP-induced apoptosis, pyroptosis and autophagy in tumor cells**

To investigate the relationship between IL10-STAT1/STAT3 signaling pathway and various programmed cell death, Calu-1 cells were treated with exogenous IL-10 protein (20 μg/mL), Fludarabine (10 μM), or Stattic (30 μM) for 12 h, respectively, before CAP treatment. As shown in Extended Figure 5, the expression levels of apoptosis protein (cleaved-PARP), pyroptosis protein (cleaved-GSDME) and autophagy protein (LC3- II/Ⅰ) in IL-10 pretreated Calu-1 cells were decreased compared to that without IL-10 treatment (Extended Figure 4A). On the contrary, the expression levels of proteins related to apoptosis, pyroptosis and autophagy were all upregulated after Fludarabine or Stattic pretreatment (Extended Figure 4B). These results indicate that IL-10-STAT1/STAT3 signaling pathway inhibited the three types of cell death induced by CAP.

**3.6. The CAP treatment had little effect on the weight of mice.**

Comparative analysis revealed that the weight of mice in PA-MCM treatment group was almost the same with that of the PAM treatment group (Extended Figure 6), indicating that CAP or macrophage had little effect on the weight of mice.

**Figure Legends**

**Extended Figure 1. Macrophages significantly reduced the killing effect of CAP on tumor cells.**

A. Viability of H1299 cells treated with CAP (30 s). B. Fraction of PI positive cells (H1299) after CAP (30 s) treatment. C. Viability of H1975 cells treated with CAP (30 s). D. Fraction of PI positive cells (H1975) after CAP (30 s). ns: no significance; *: *p* < 0.05; **: *p* < 0.01; ***: *p* < 0.001.

**Extended Figure 2. The induction of macrophages polarization.**

A. Morphological changes of macrophages after CAP treatment. B. Typical results of flow cytometry for detecting macrophage polarization. C&D. The fraction of CD80 (C) and CD206 (D) positive macrophages after LPS + IFN-γ treatment. E. Typical results of flow cytometry for detecting macrophage polarization. F&G. The fraction of CD80 (F) and CD206 (G) positive macrophages after IL-4 + IL-13 treatment. ns: no significance; *: *p* < 0.05; **: *p* < 0.01.

**Extended Figure 3. Macrophages attenuated the killing effect of CAP through IL-10/STAT1/STAT3 pathway.**

The expression level of IL-10 in the culture supernatants of THP-1, ***: *p*<0.001.

**Extended Figure 4. Macrophages suppressed CAP-induced apoptosis, pyroptosis and autophagy in tumor cells via alleviating ER stress**

Effect of NH_4_Cl (100 mM) on the viability of Calu-1 cells after CAP treatment. *: *p* < 0.05.

**Extended Figure 5. IL-10-STAT1/STAT3 inhibit CAP-induced apoptosis, pyroptosis and autophagy in tumor cells.**

Effect of IL-10 (20 μg/mL) (A), Fludarabine (10 mM) (B) and Stattic (30 mM) (C) on the expression of apoptosis, pyroptosis and autophagy-related proteins in Calu-1 cells after CAP treatment.

**Extended Figure 6. The CAP treatment had little effect on the weight of mice.**
