# Supplementary material for: M2 polarization of macrophage protects the lung cancer cells from cold atmospheric plasma via alleviating endoplasmic reticulum stress
Source: Cell Death Discov. 2025 Oct 27;11:487. doi: 10.1038/s41420-025-02775-4 (PMC12559389; doi:10.1038/s41420-025-02775-4)
Supplement: Supplementary file 3 — Extended Table-2 [file 41420_2025_2775_MOESM3_ESM.docx]

Extended Table 2

| Liquid type | Preparation method |
| --- | --- |
| PAM | the RPMI-1640 medium treated by CAP for 30 s. |
| MCM | the conditioned medium collected from the cultured M0 macrophages after 24 h incubation. |
| PAM-24 h | the plasma-activated RPMI-1640 medium placed in the cell incubator for 24 h was labeled as PAM-24 h to distinguish from the freshly made PAM. |
| PA-MCM | the M0 macrophages incubated with fresh PAM (10 mL) for 24 h, and the supernatant was collected and labelled as PA-MCM. |
