# Supplementary material for: M2 polarization of macrophage protects the lung cancer cells from cold atmospheric plasma via alleviating endoplasmic reticulum stress
Source: Cell Death Discov. 2025 Oct 27;11:487. doi: 10.1038/s41420-025-02775-4 (PMC12559389; doi:10.1038/s41420-025-02775-4)
Supplement: Supplementary file 10 — WB Original data [file 41420_2025_2775_MOESM10_ESM.pdf]

Fig 3E (Calu-1)

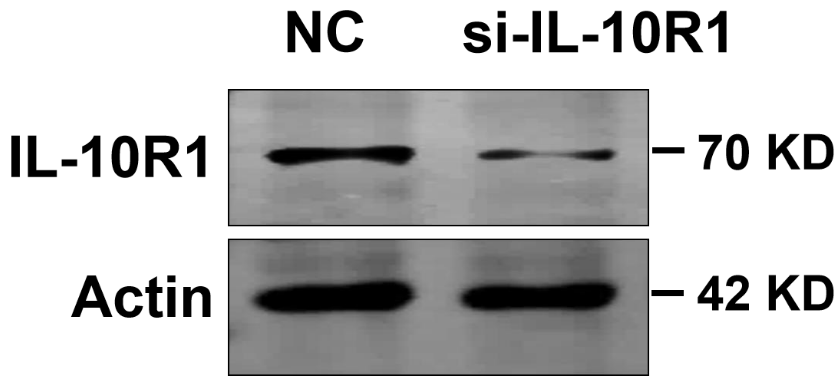

Fig 3F. The protein expression levels of IL-10R1 in Calu-1 cells after siRNA treatment.

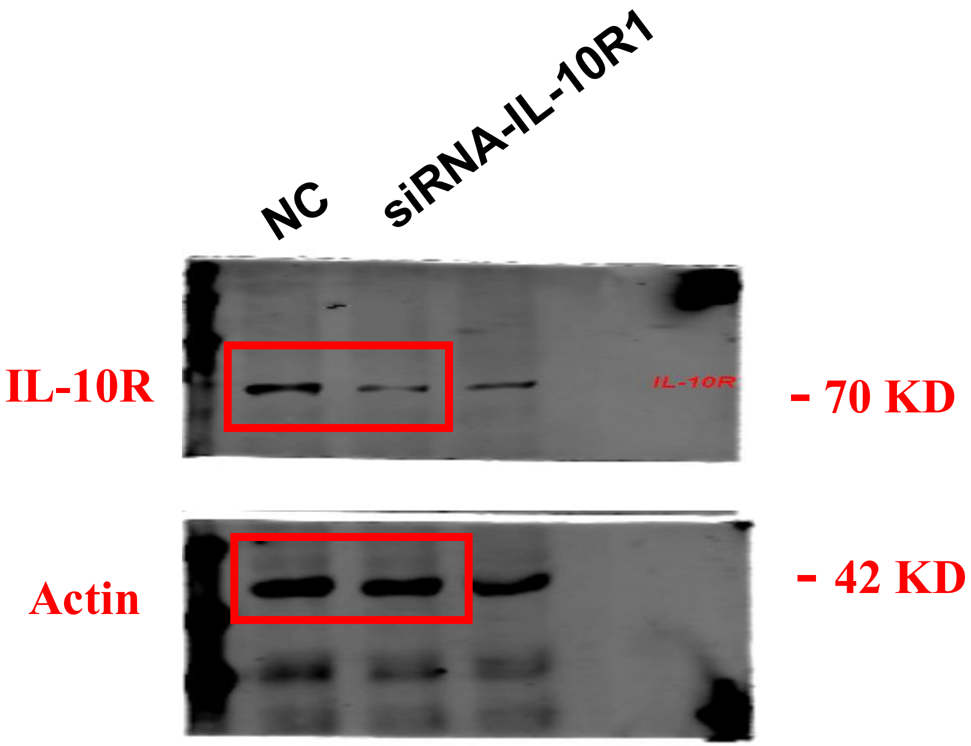

Fig 3G (Calu-1)

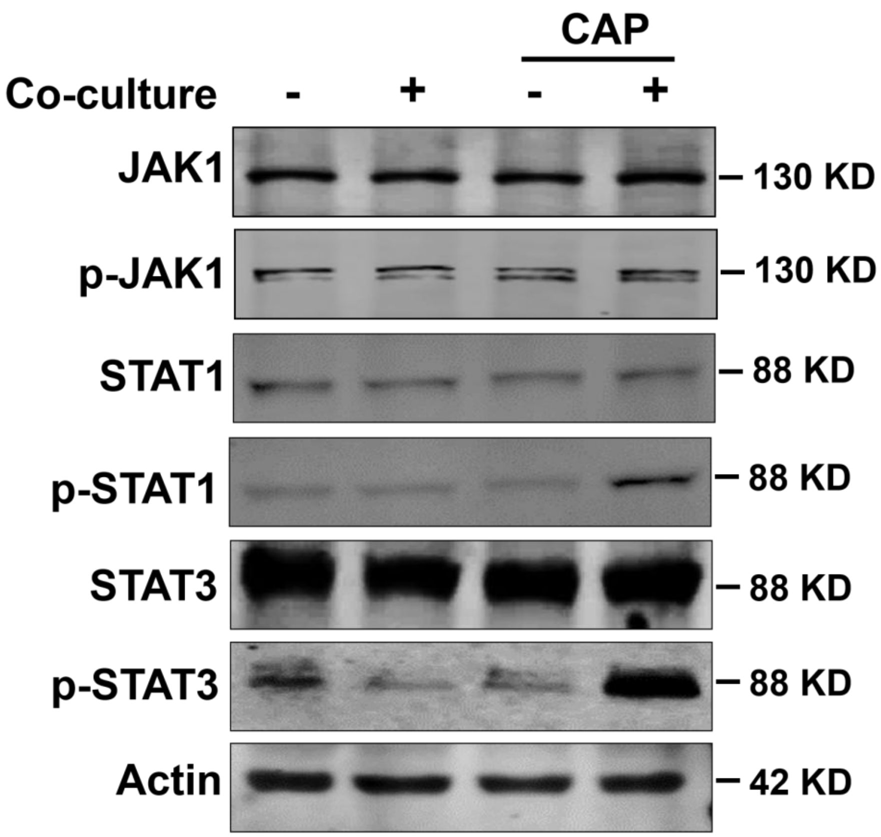

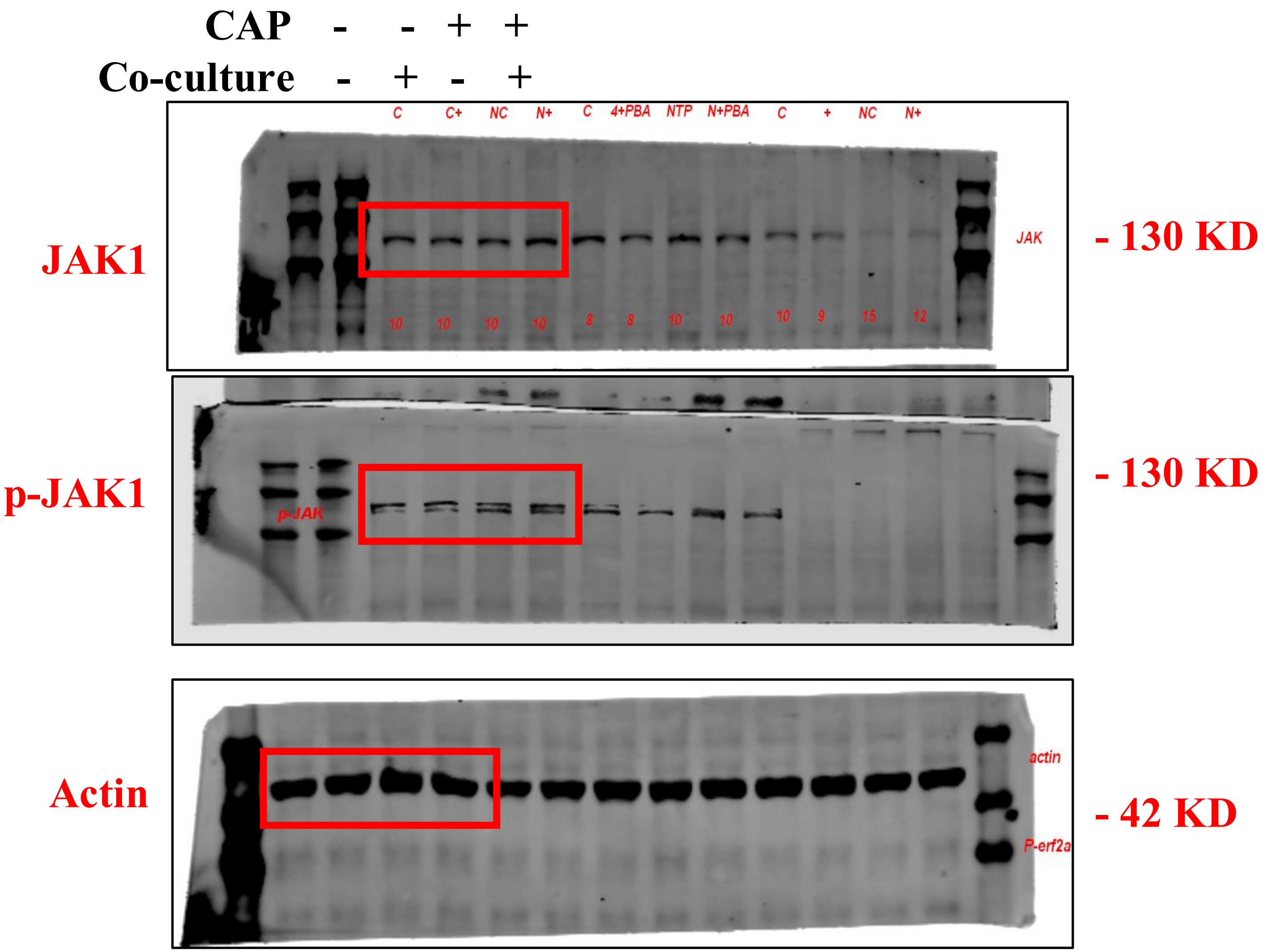

Fig 3G. The protein expression levels of JAK1, p-JAK1, STAT1, p-STAT1, STAT3, p-STAT3 and Actin in Calu-1 cells after CAP treatment.

Fig 3G (Calu-1)

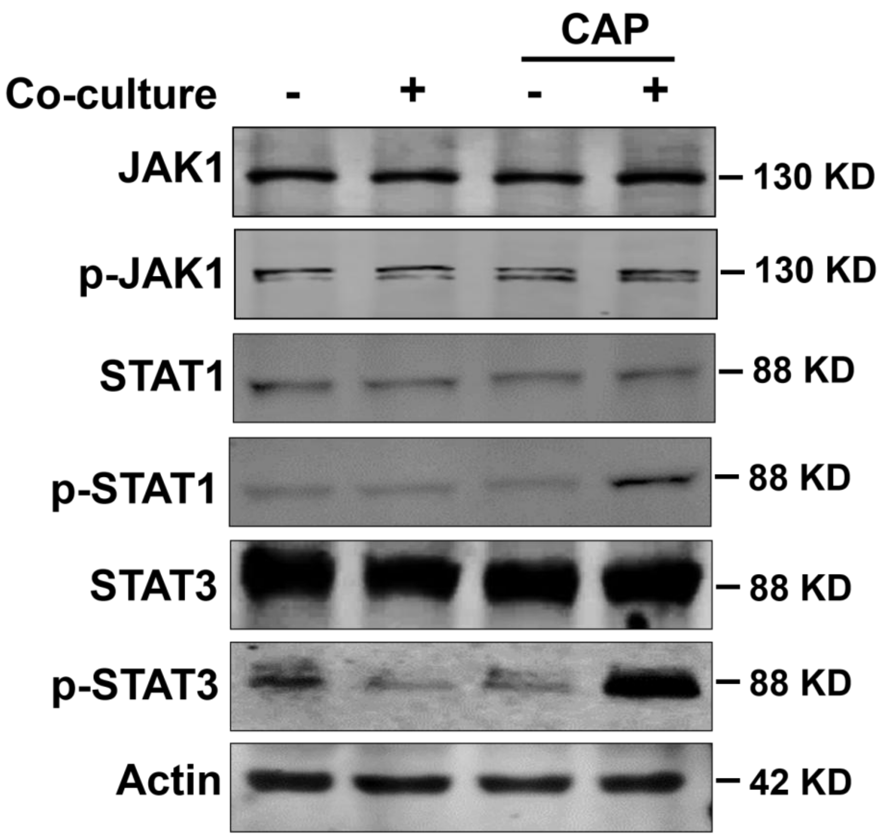

Fig 3G. The protein expression levels of JAK1, p-JAK1, STAT1, p-STAT1, STAT3, p-STAT3 and Actin in Calu-1 cells after CAP treatment.

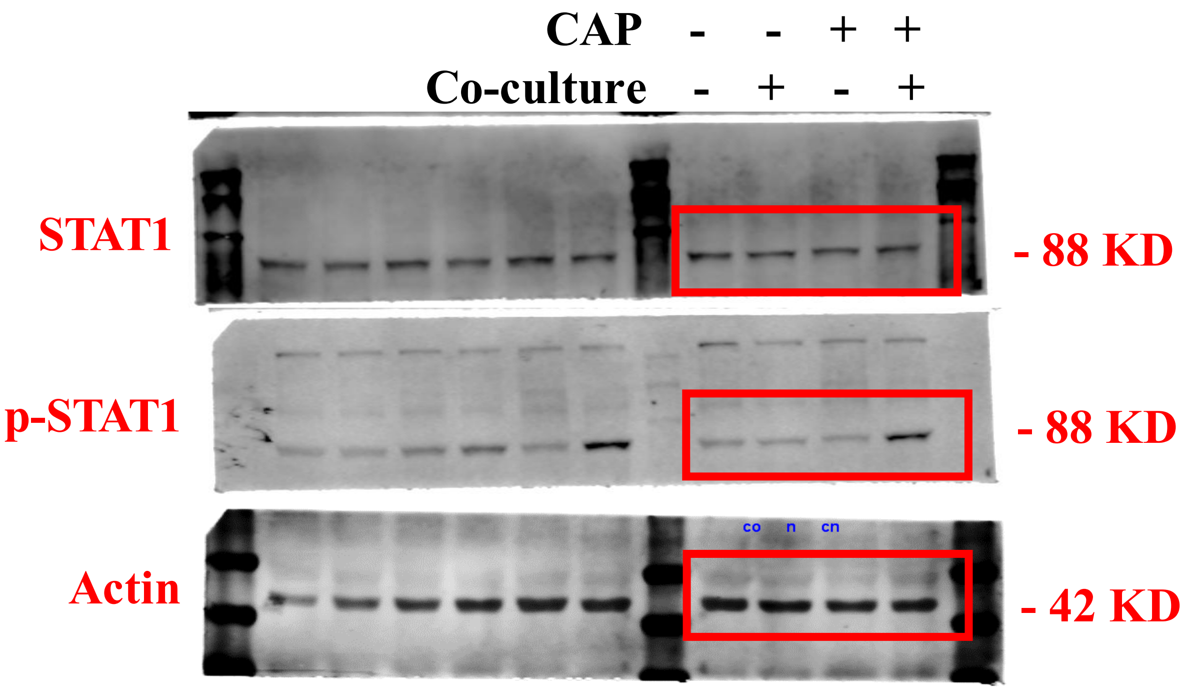

Fig 3G (Calu-1)

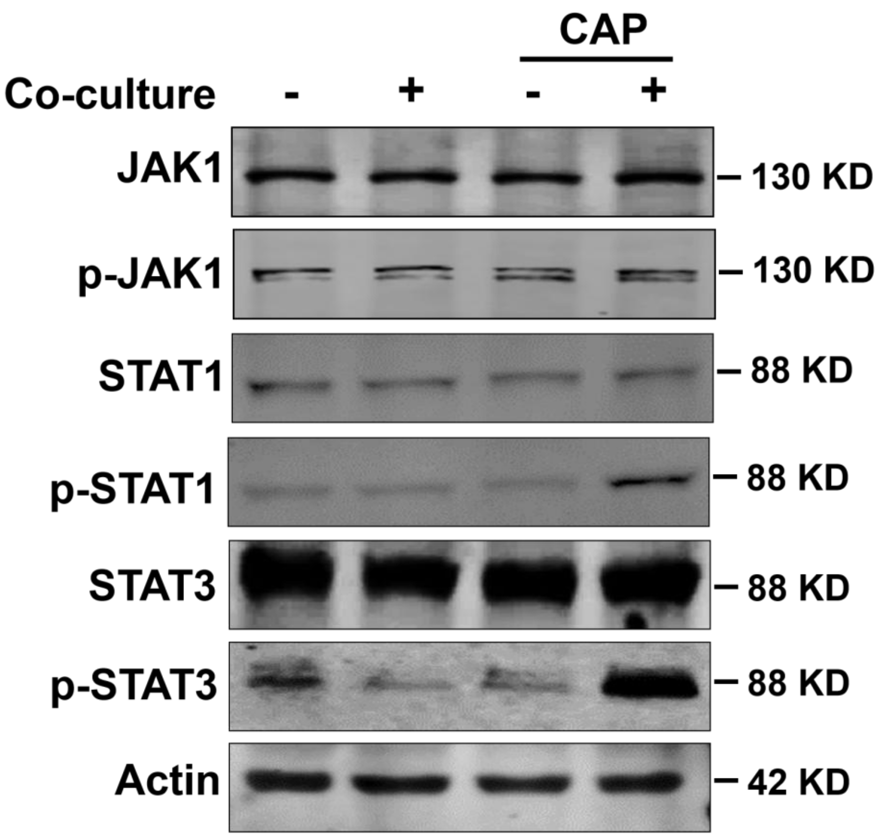

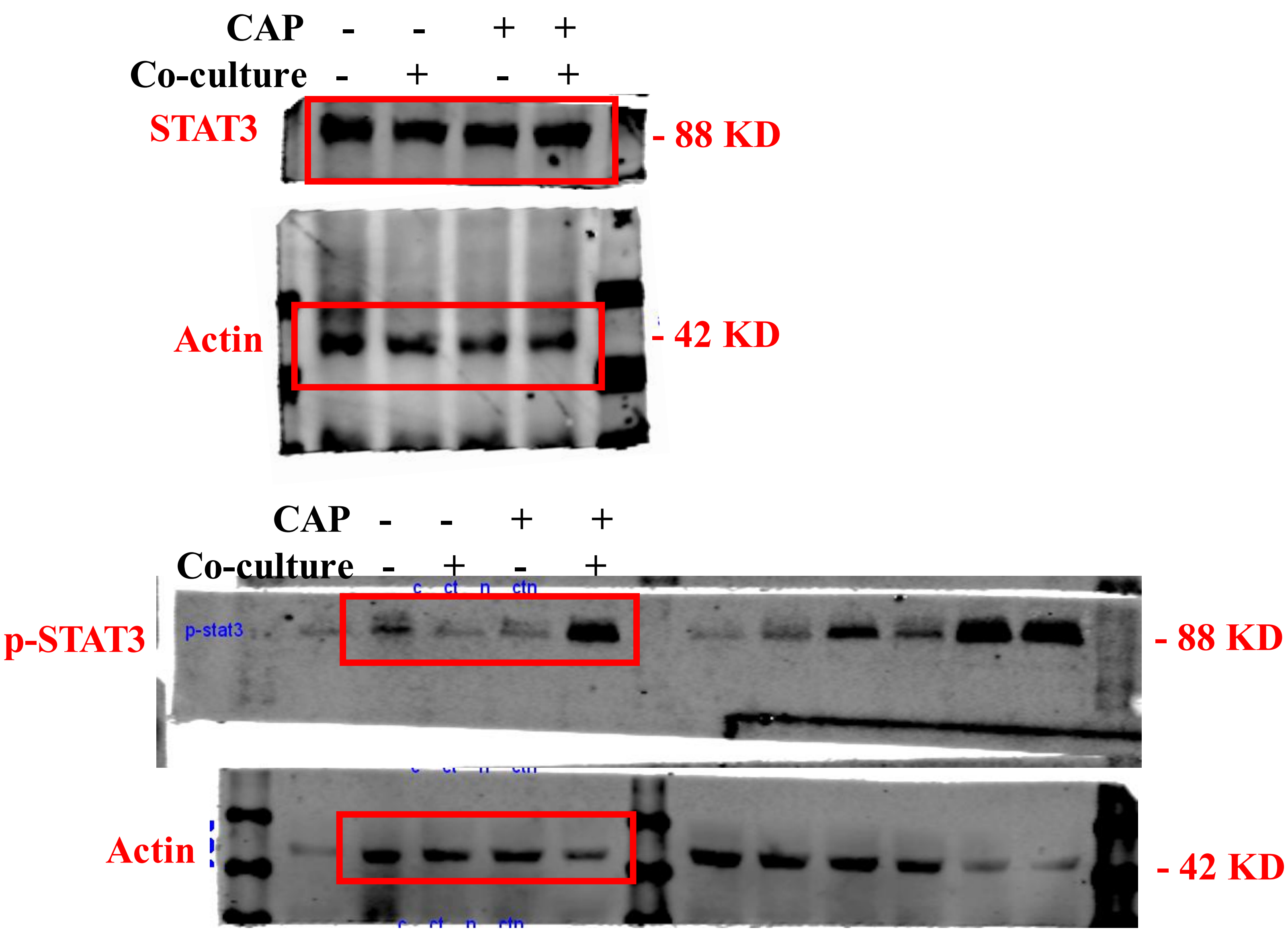

Fig 3G. The protein expression levels of JAK1, p-JAK1, STAT1, p-STAT1, STAT3, p-STAT3 and Actin in Calu-1 cells after CAP treatment.

Fig 3H (Calu-1)

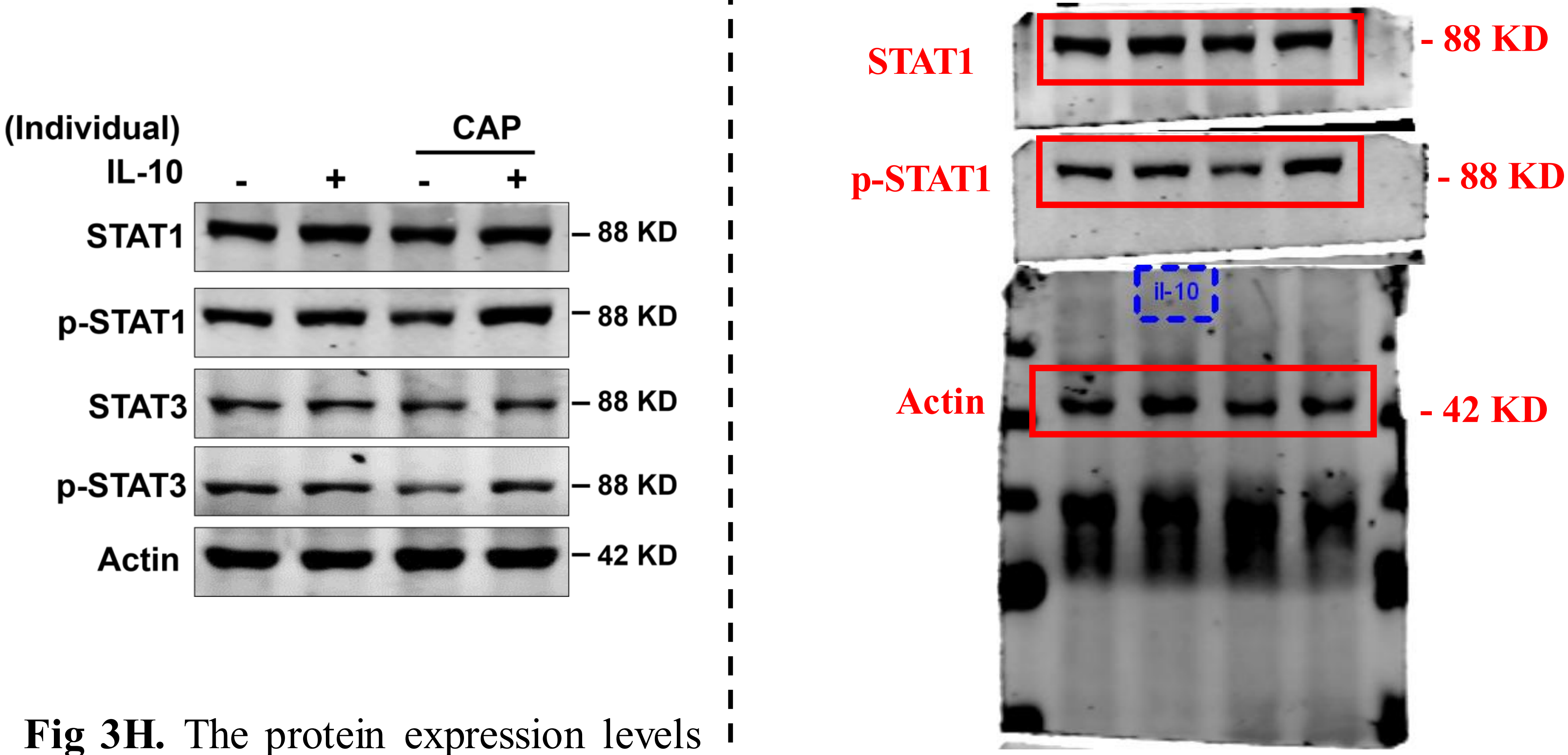

Fig 3H. The protein expression levels of STAT1, p-STAT1, STAT3, p-STAT3 and Actin in Calu-1 cells after CAP and IL-10 treatment.

Fig 3H (Calu-1)

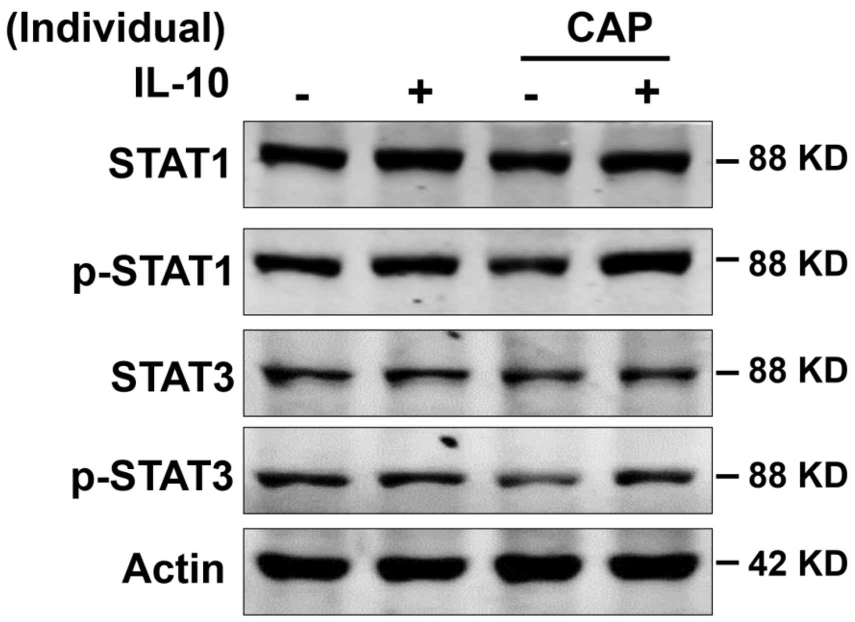

Fig 3H. The protein expression levels of STAT1, p-STAT1, STAT3, p-STAT3 and Actin in Calu-1 cells after CAP and IL-10 treatment.

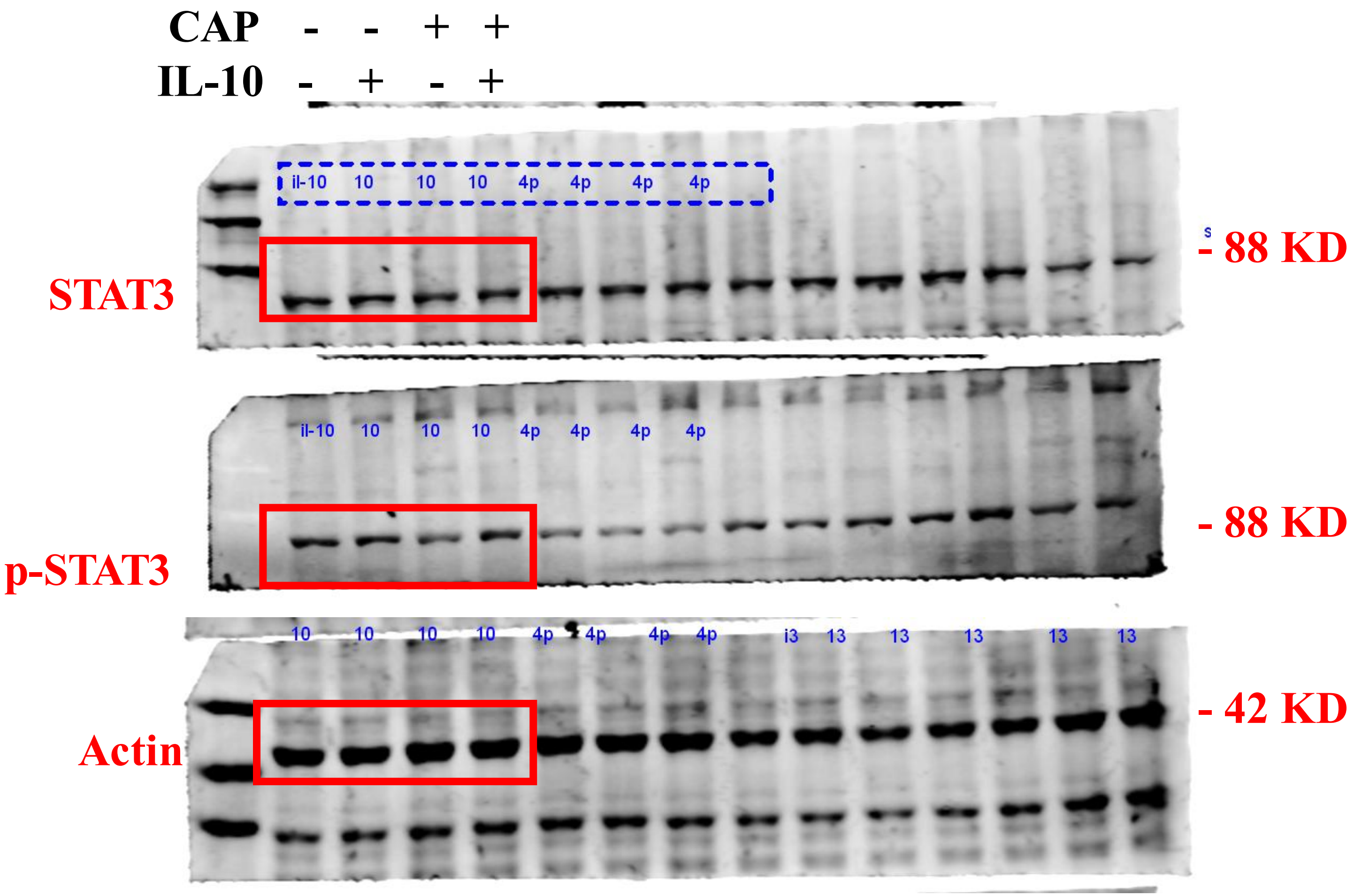

Fig 3I (Calu-1)

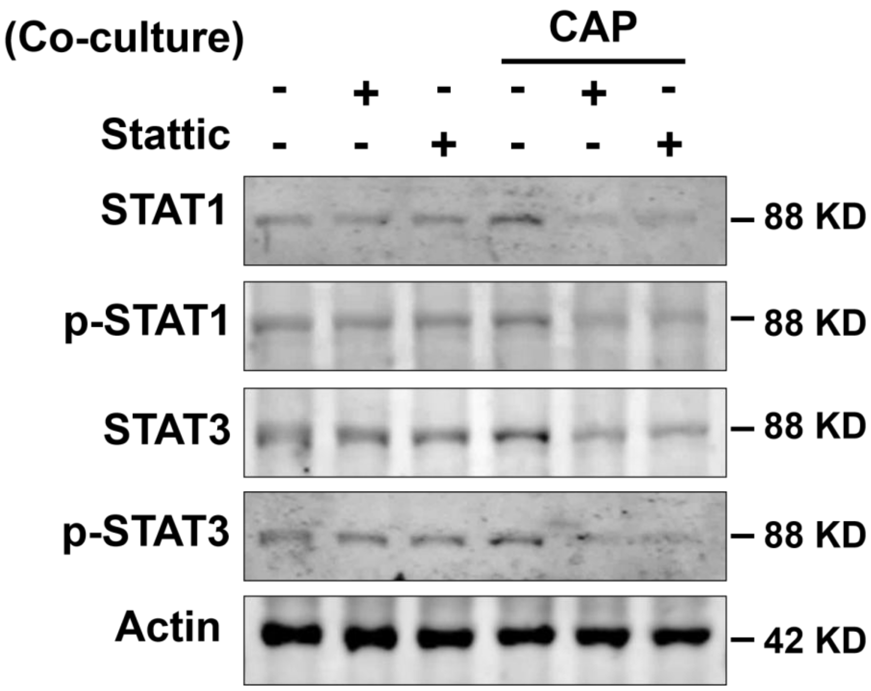

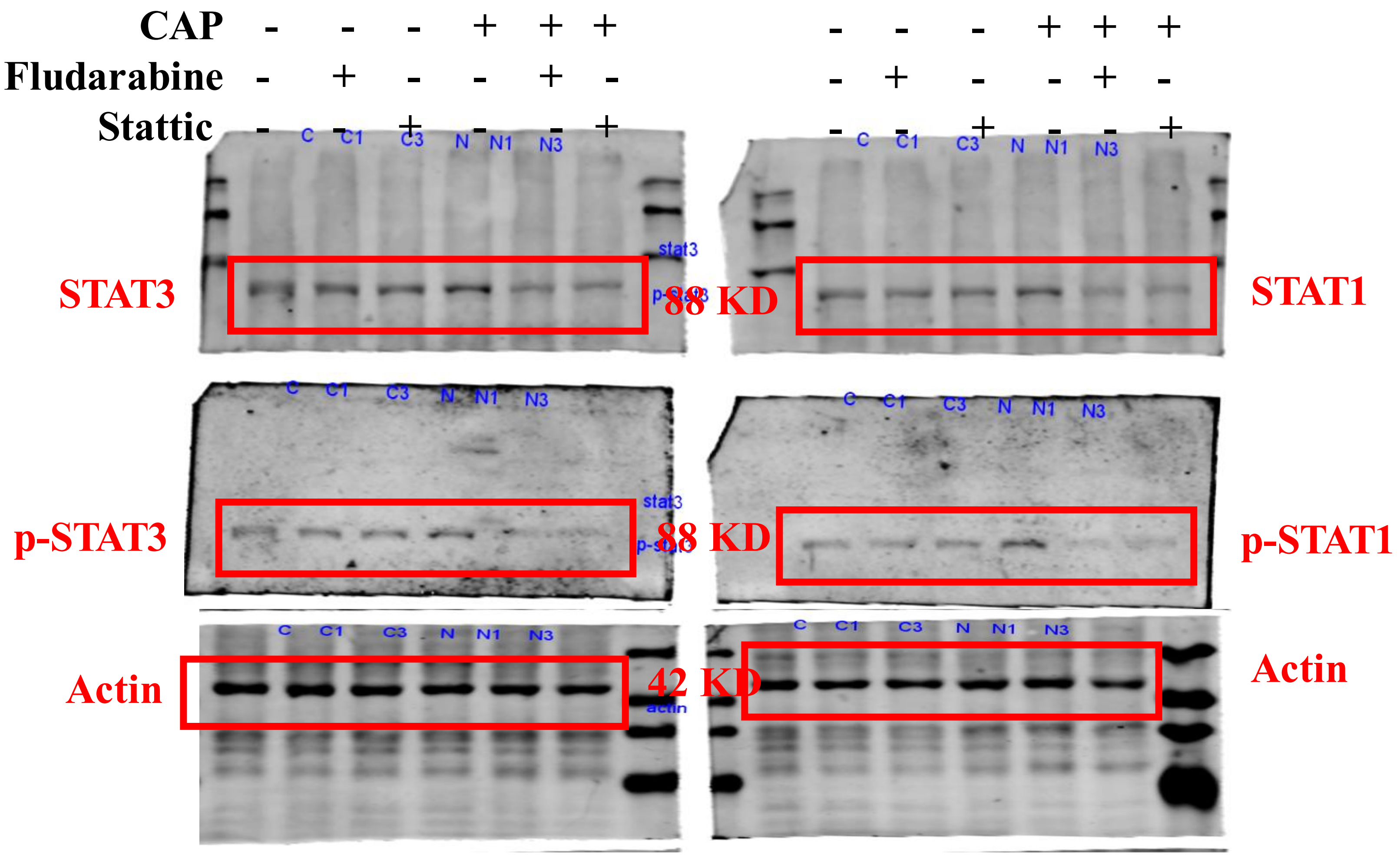

Fig 3I. The protein expression levels of STAT1, p-STAT1, STAT3, p-STAT3 and Actin in Calu-1 cells after CAP and Fludarabine , Stattic treatment.

Fig 4G (Calu-1)

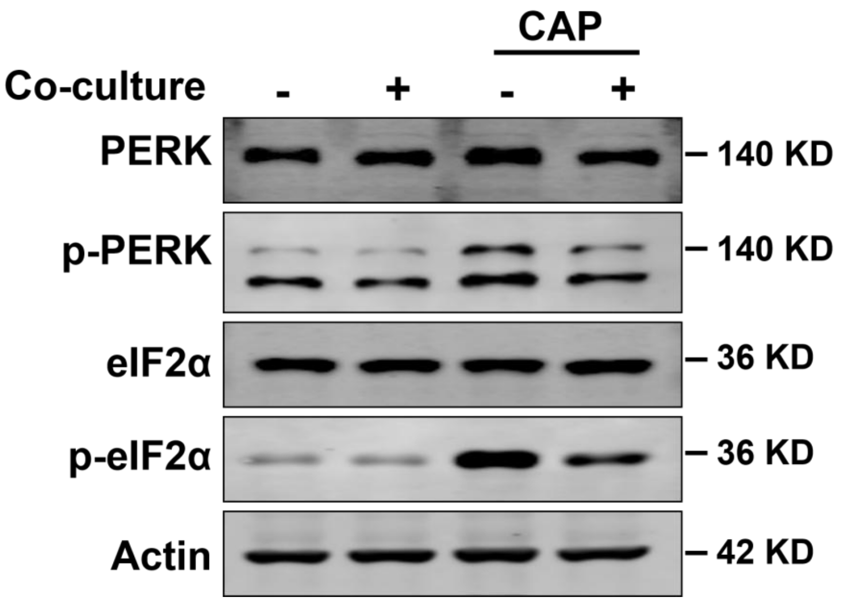

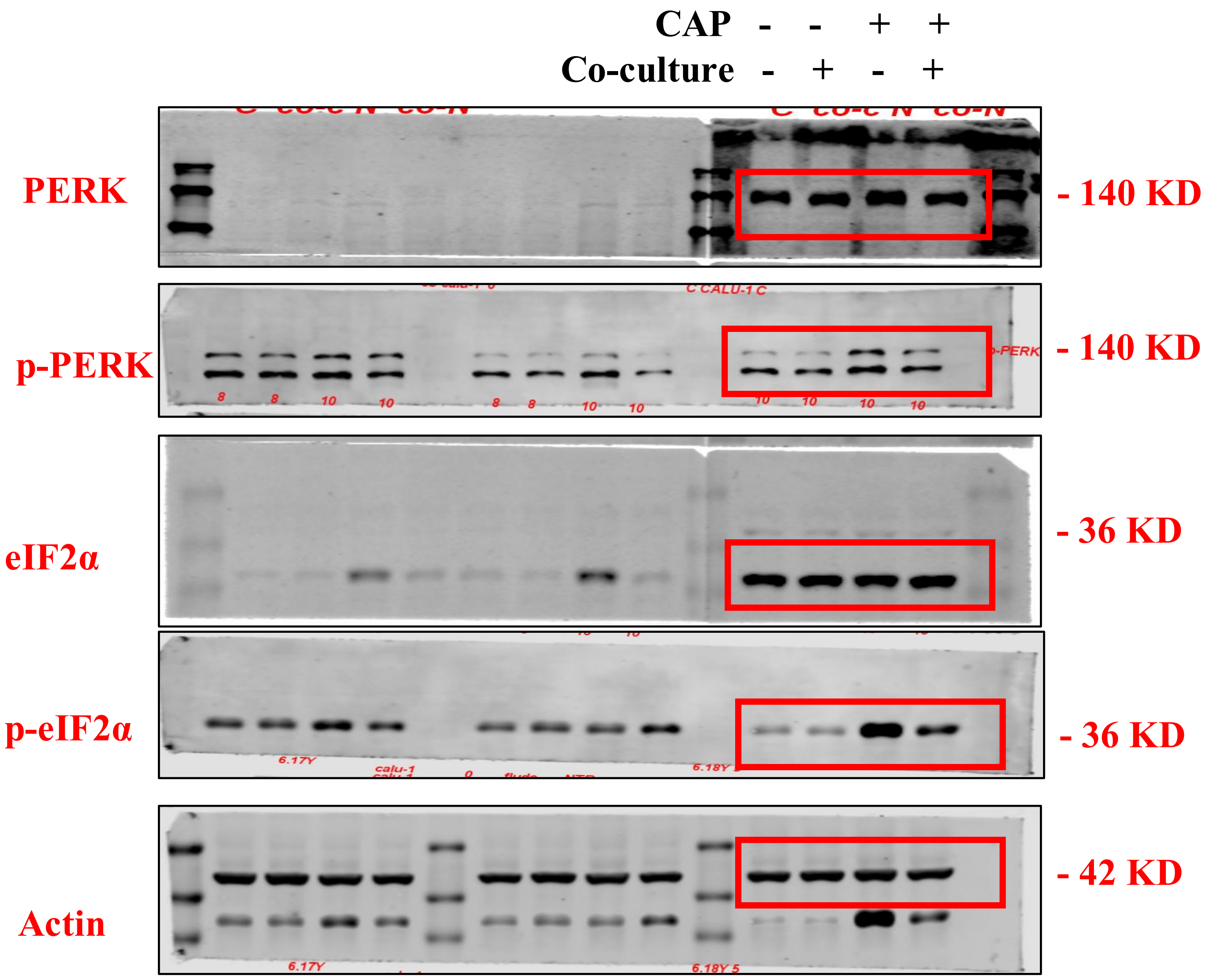

Fig 4G. The protein expression levels of PERK, p-PERK, eIF2α and p-eIF2α in Calu-1 cells after CAP treatment.

Fig 4H (Calu-1)

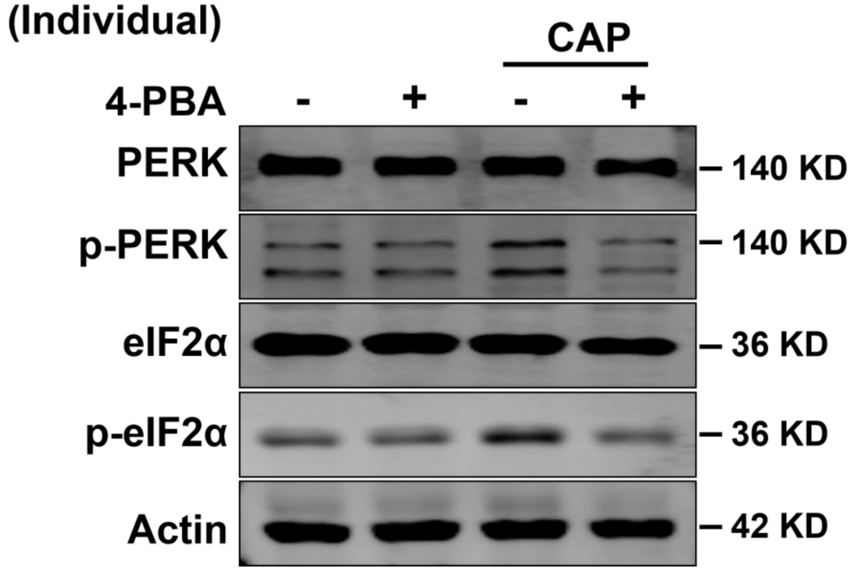

Fig 4H. The protein expression levels of PERK, p-PERK, eIF2α and p-eIF2α in Calu-1 cells after CAP and 4-PBA treatment.

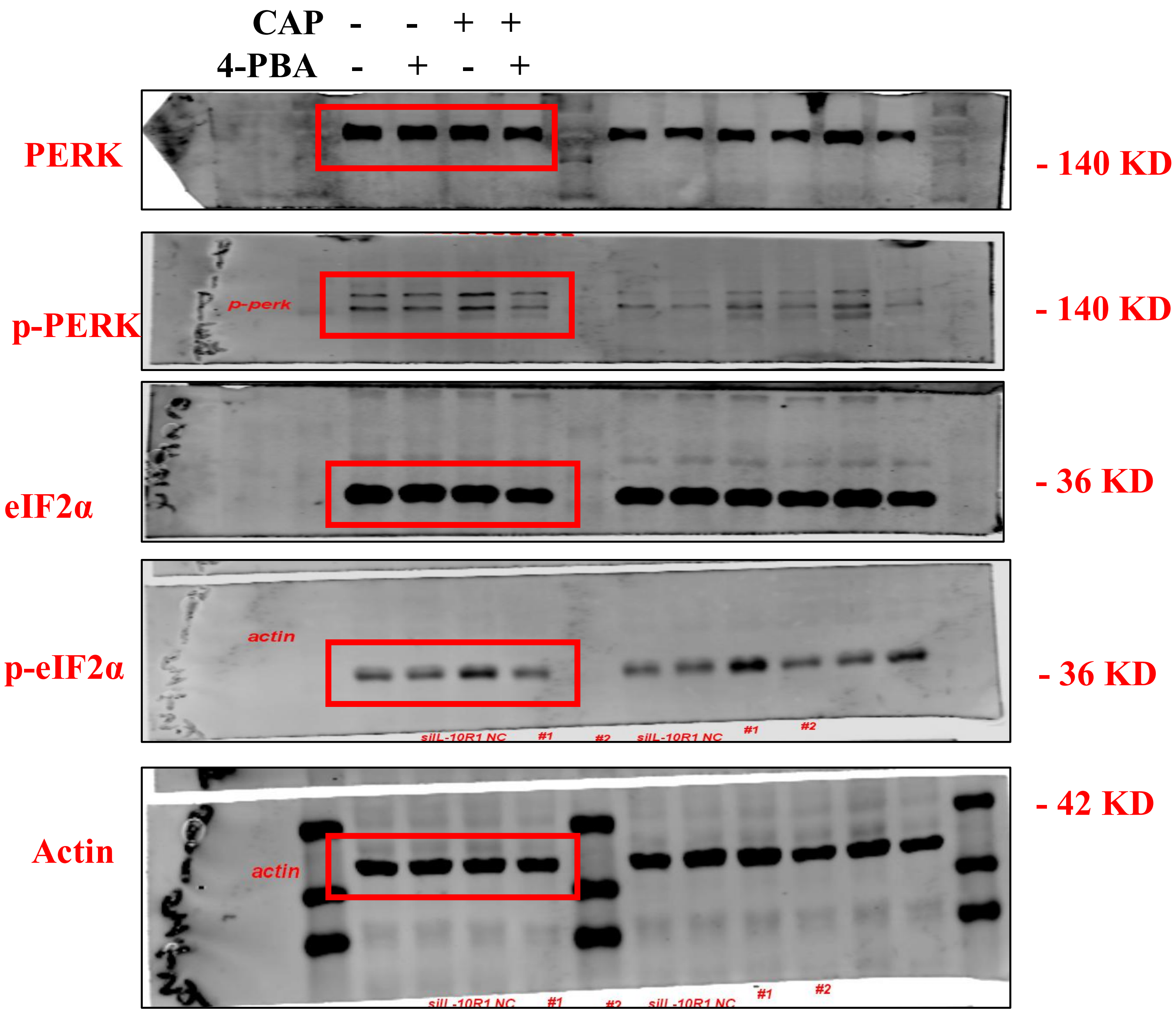

Fig 4J (Calu-1)

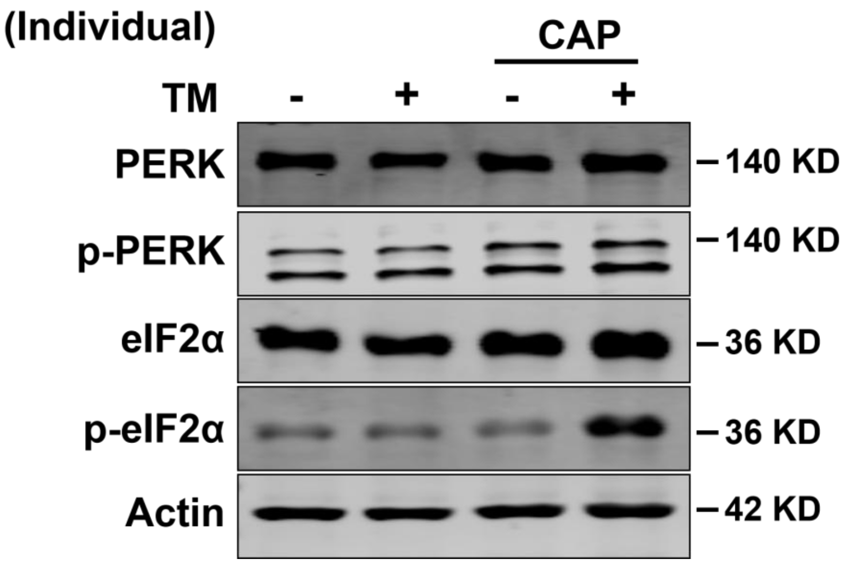

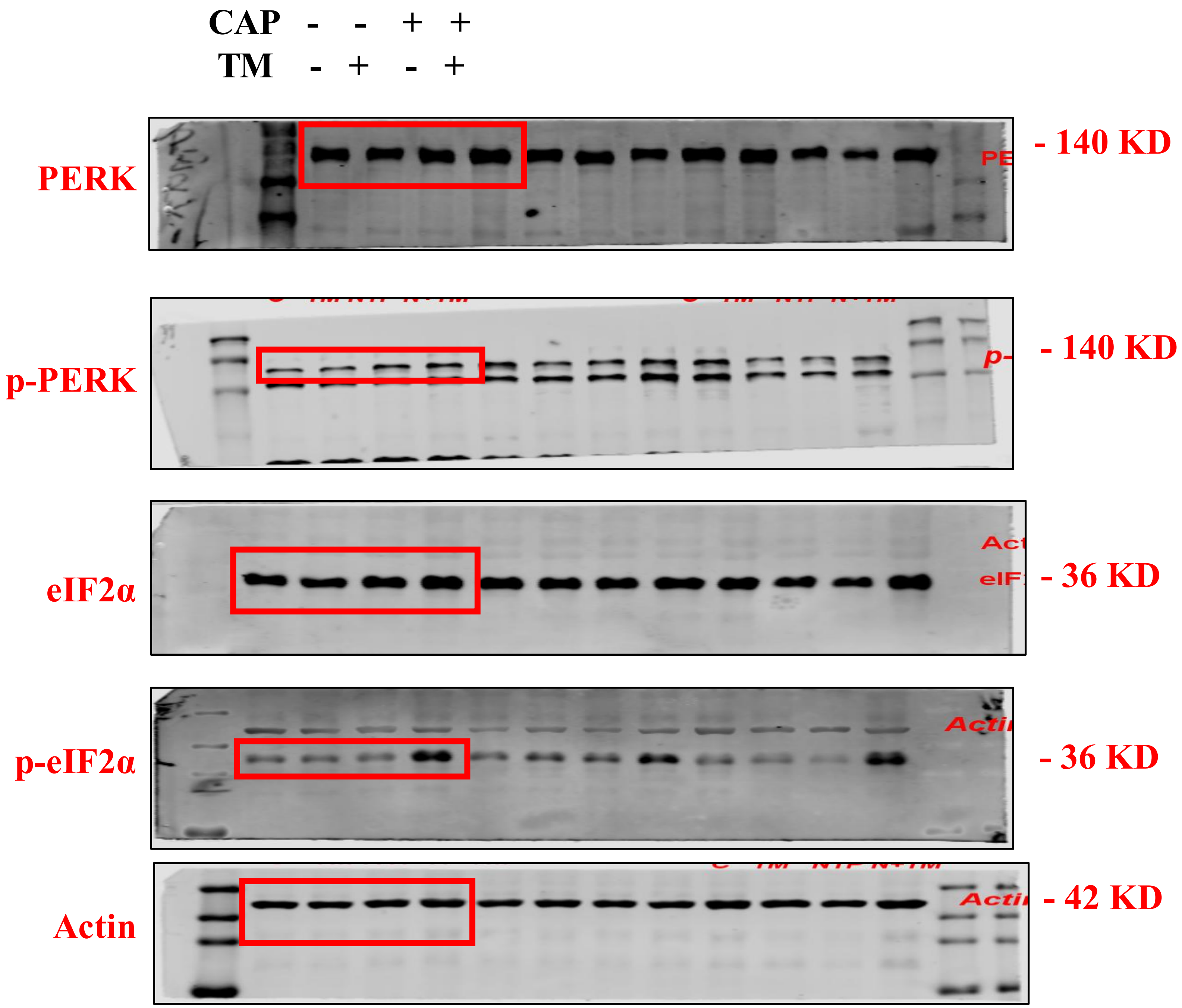

Fig 4J. The protein expression levels ofPERK, p-PERK, eIF2α and p-eIF2α in Calu-1 cells after CAP and TM treatment.

Fig 4L (Calu-1)

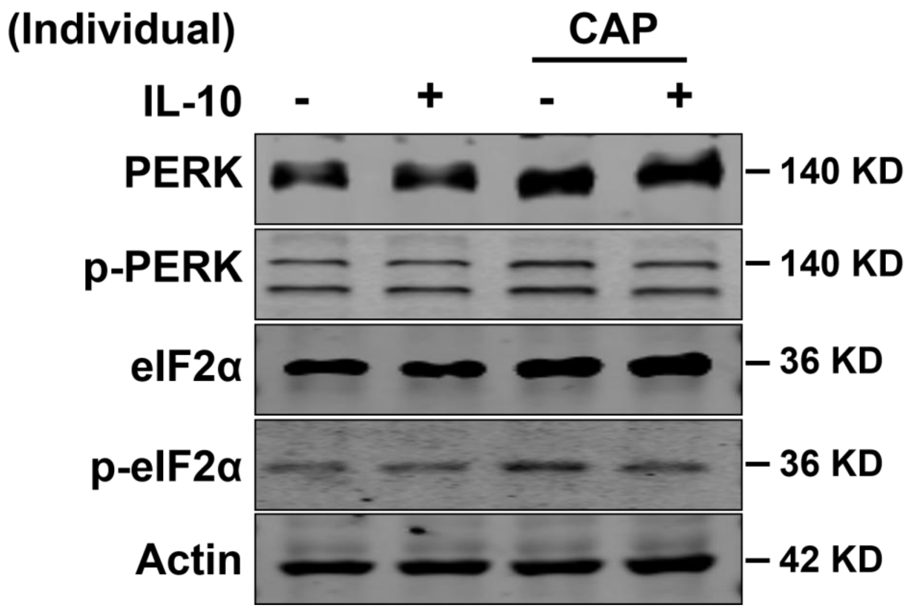

Fig 4L. The protein expression levels of PERK, p-PERK, eIF2α and p-eIF2α in Calu-1 cells after CAP and IL-10 treatment.

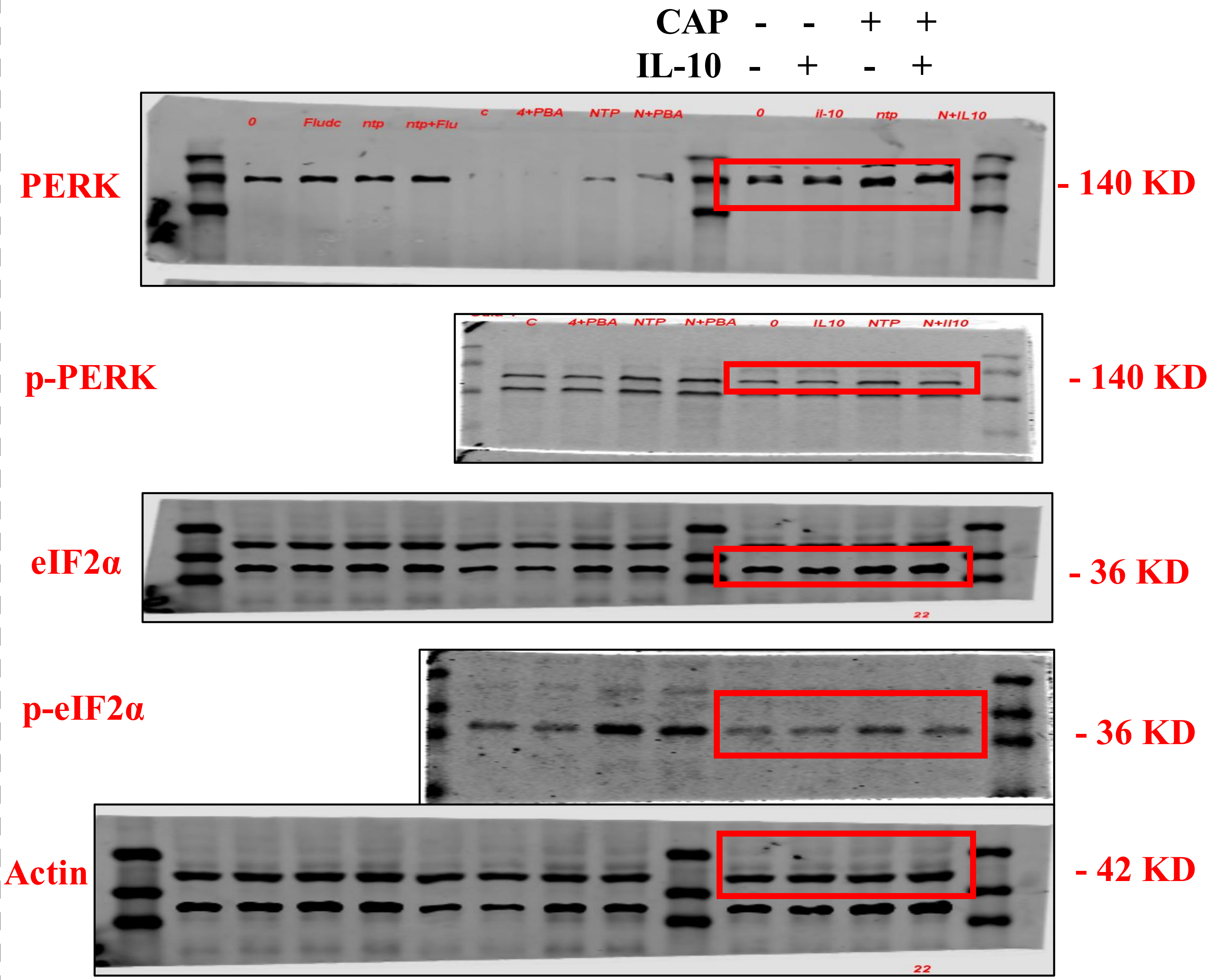

Fig 4M (Calu-1)

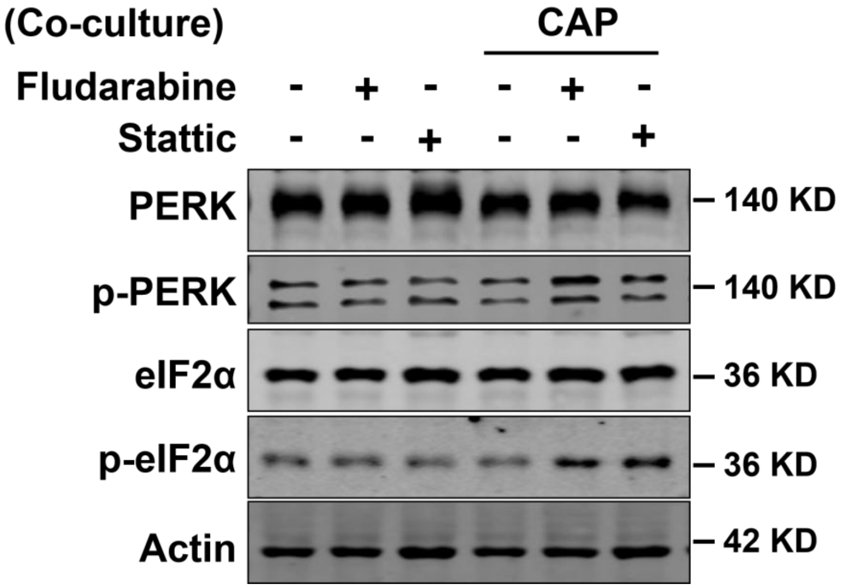

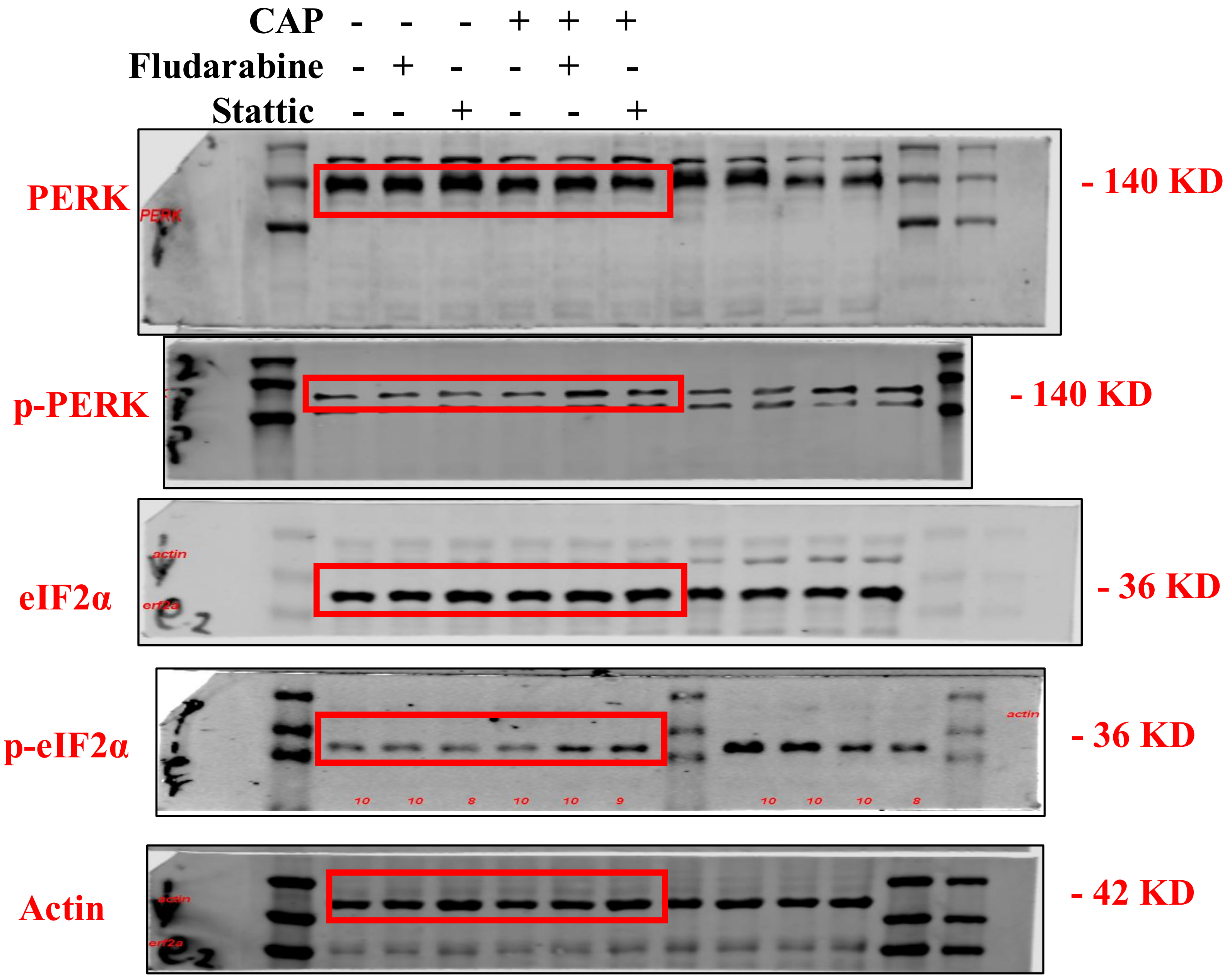

Fig 4M. The protein expression levels of PERK, p-PERK, eIF2α and p-eIF2α in Calu-1 cells after CAP and Fludarabine, Stattic treatment.

Fig 5A (Calu-1)

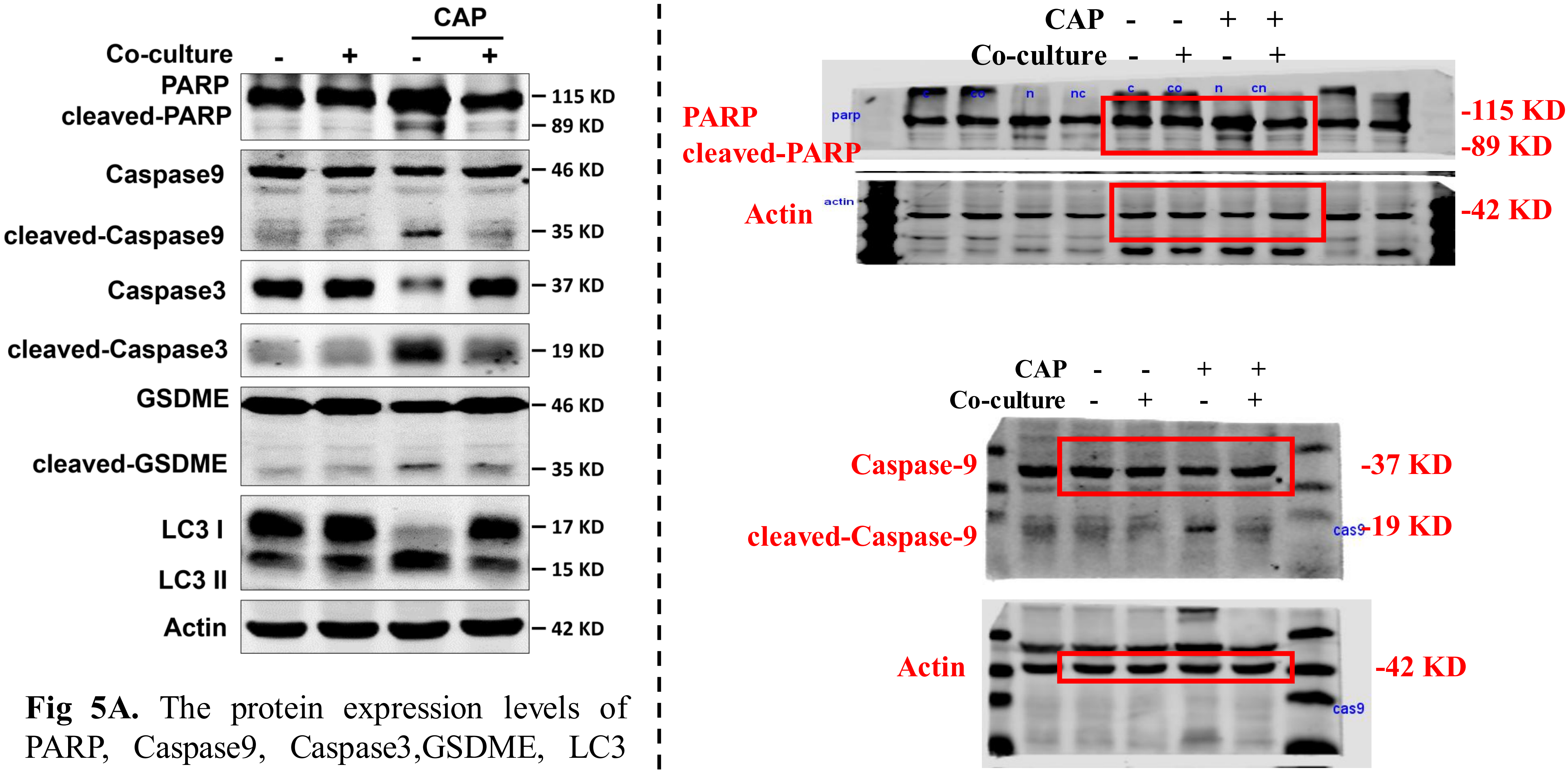

Fig 5A. The protein expression levels of PARP, Caspase9, Caspase3,GSDME, LC3 and Actin in Calu-1 cells after CAP treatment.

Fig 5A (Calu-1)

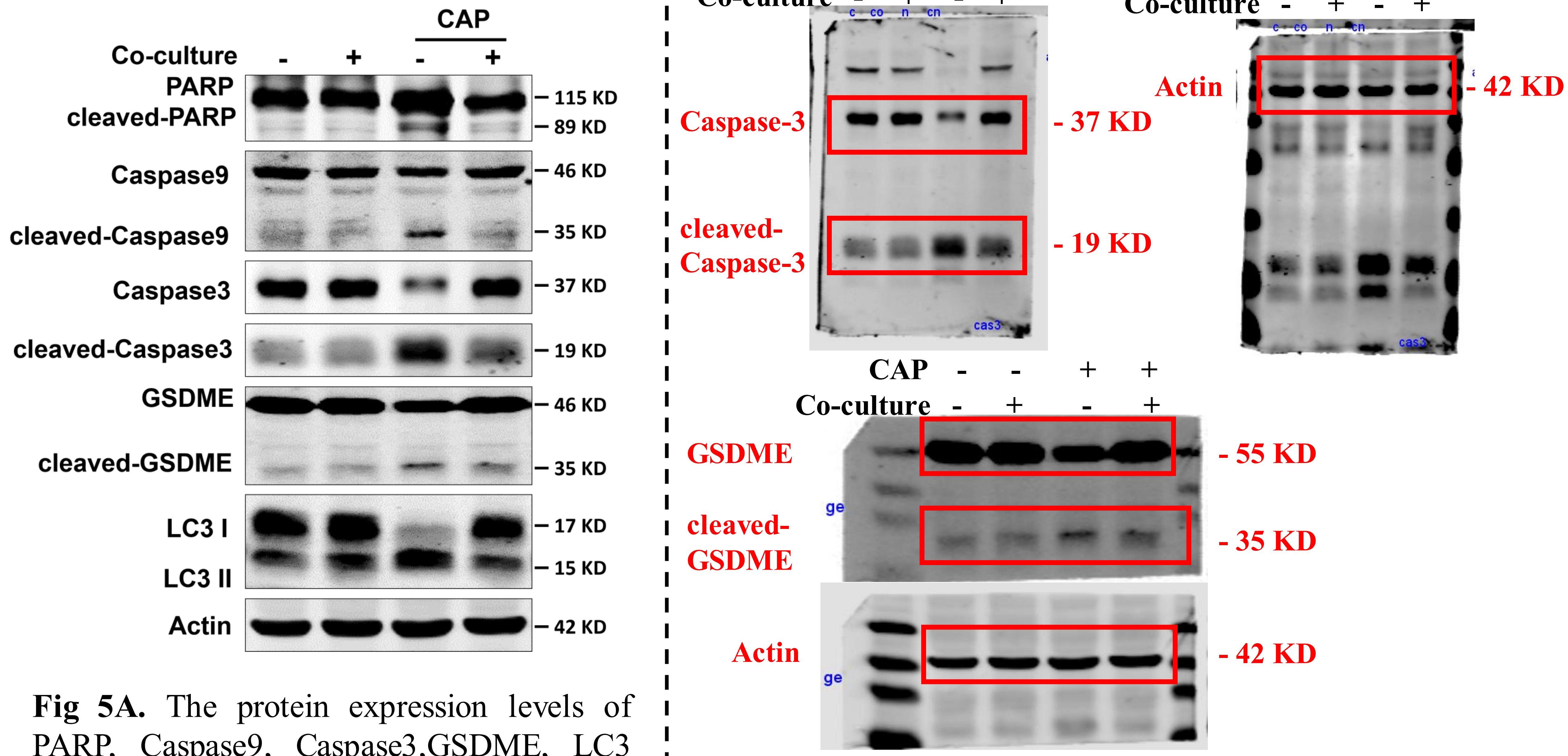

Fig 5A. The protein expression levels of PARP, Caspase9, Caspase3,GSDME, LC3 and Actin in Calu-1 cells after CAP treatment.

Fig 5A (Calu-1)

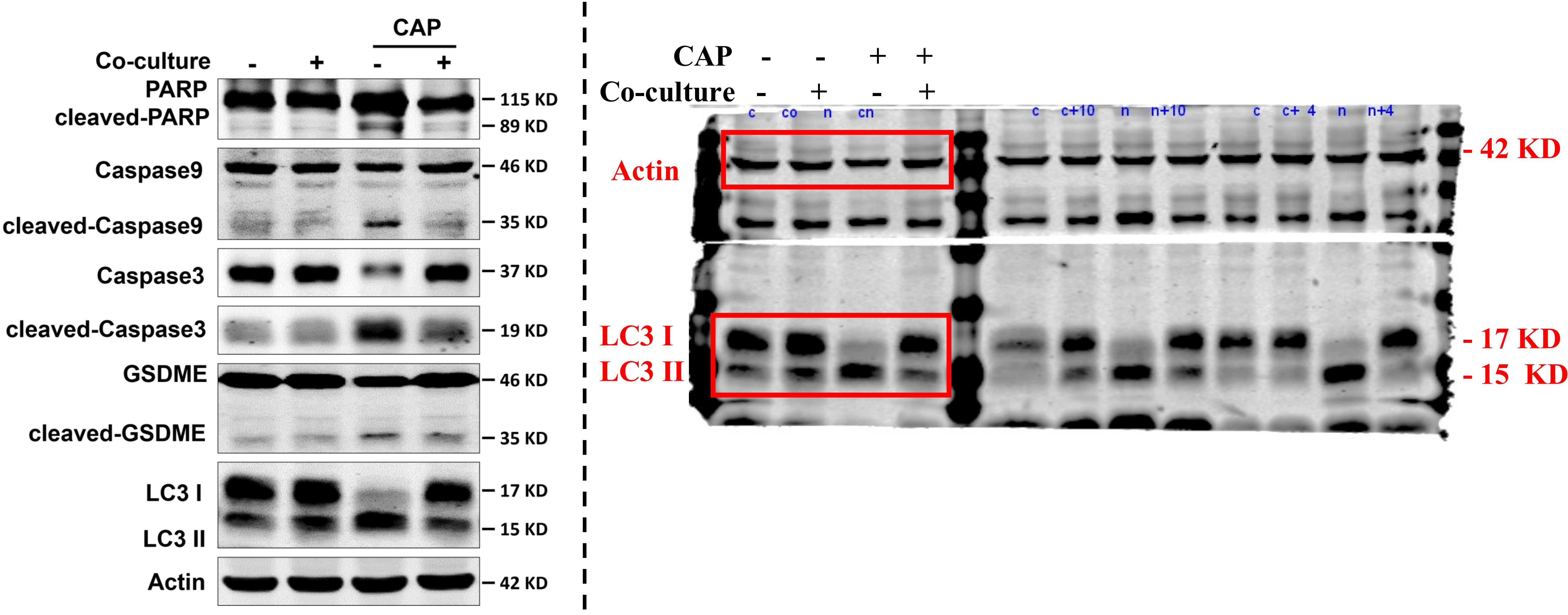

Fig 5A. The protein expression levels of PARP, Caspase9, Caspase3,GSDME, LC3 and Actin in Calu-1 cells after CAP treatment.

Fig 5B (Calu-1)

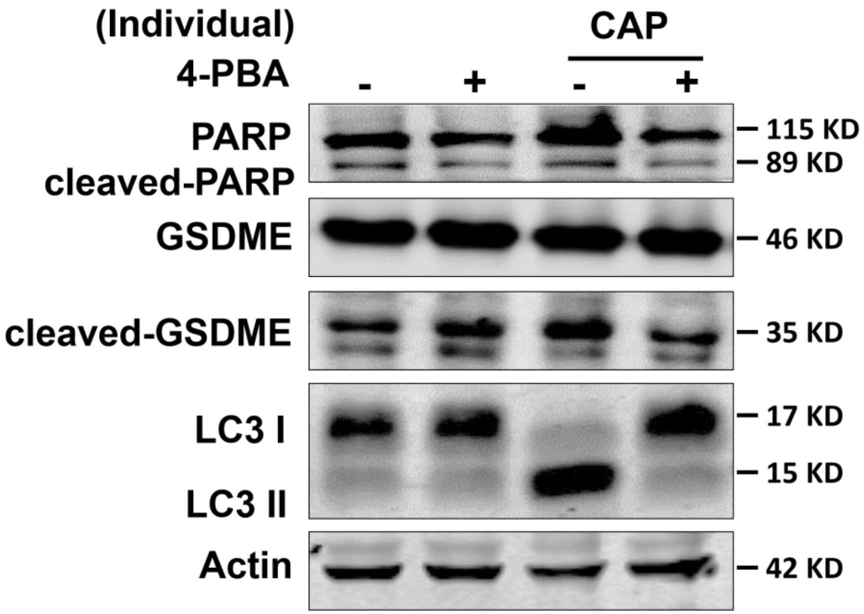

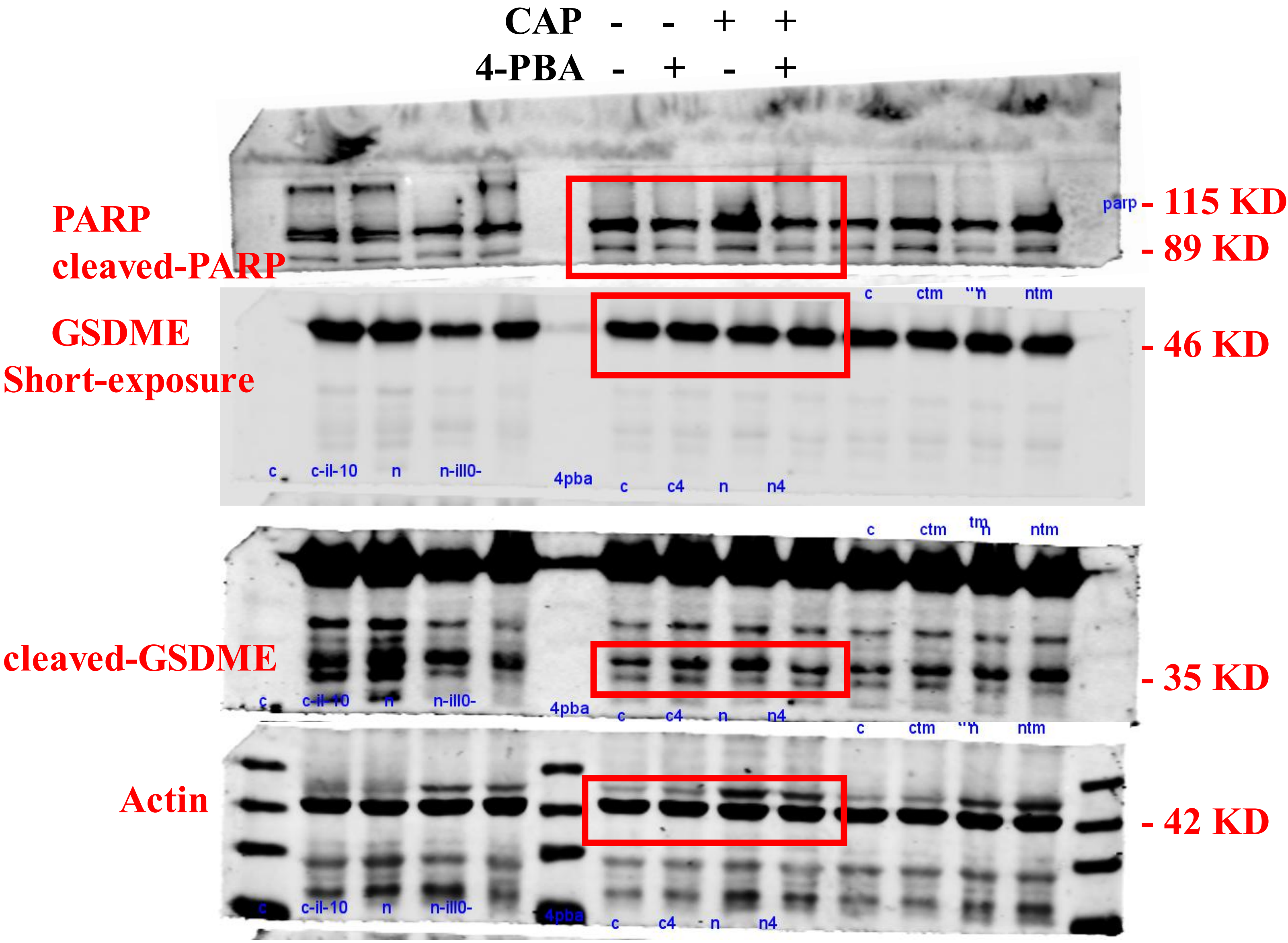

Fig 5B. The protein expression levels of PARP, Caspase9, Caspase3,GSDME, LC3 and Actin in Calu-1 cells after CAP and 4-PBA treatment.

Fig 5B (Calu-1)

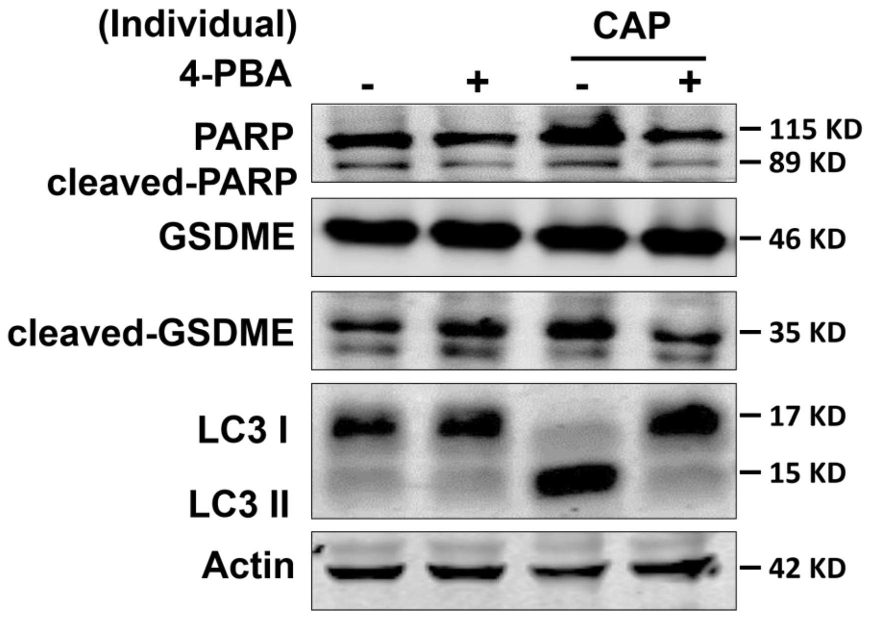

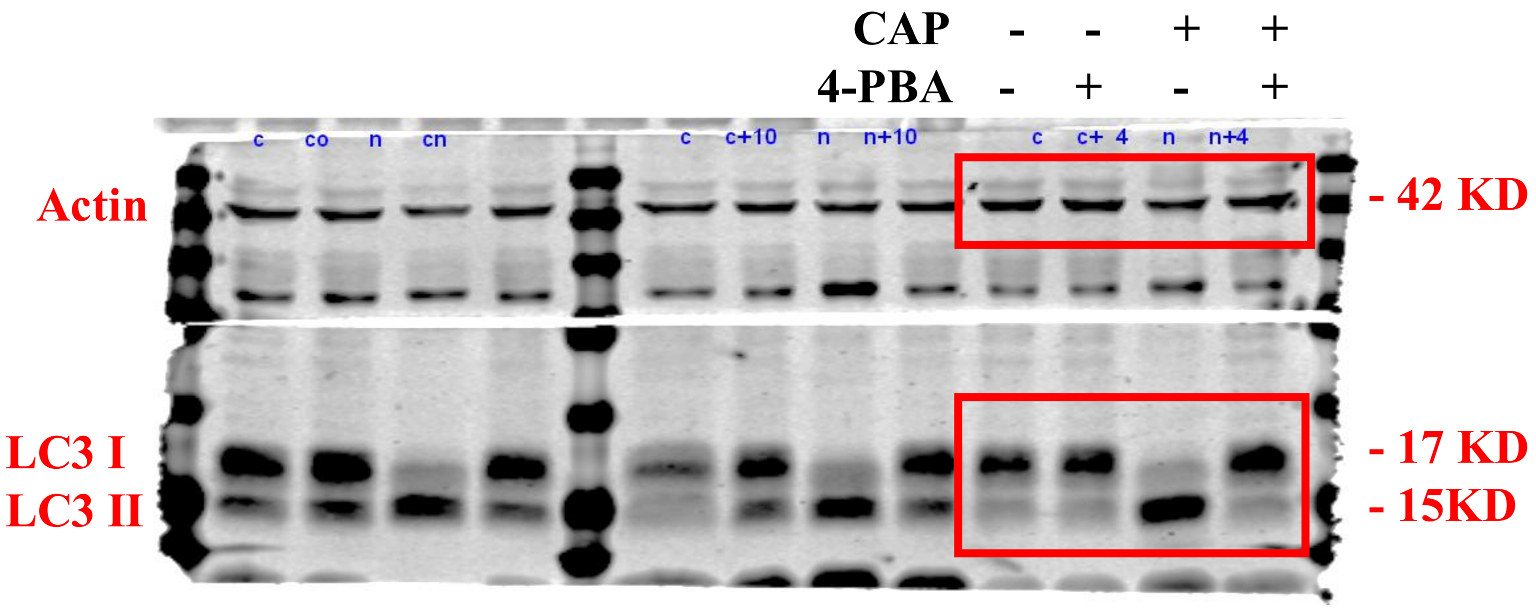

Fig 5B. The protein expression levels of PARP, Caspase9, Caspase3,GSDME, LC3 and Actin in Calu-1 cells after CAP and 4-PBA treatment.

Fig 5C (Calu-1)

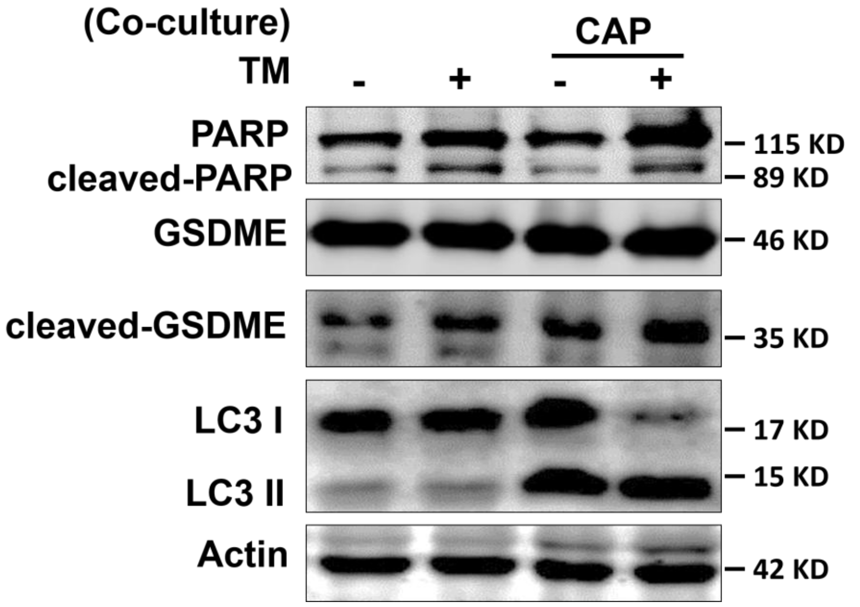

Fig 5C. The protein expression levels of PARP, Caspase9, Caspase3,GSDME, LC3 and Actin in Calu-1 cells after CAP and TM treatment.

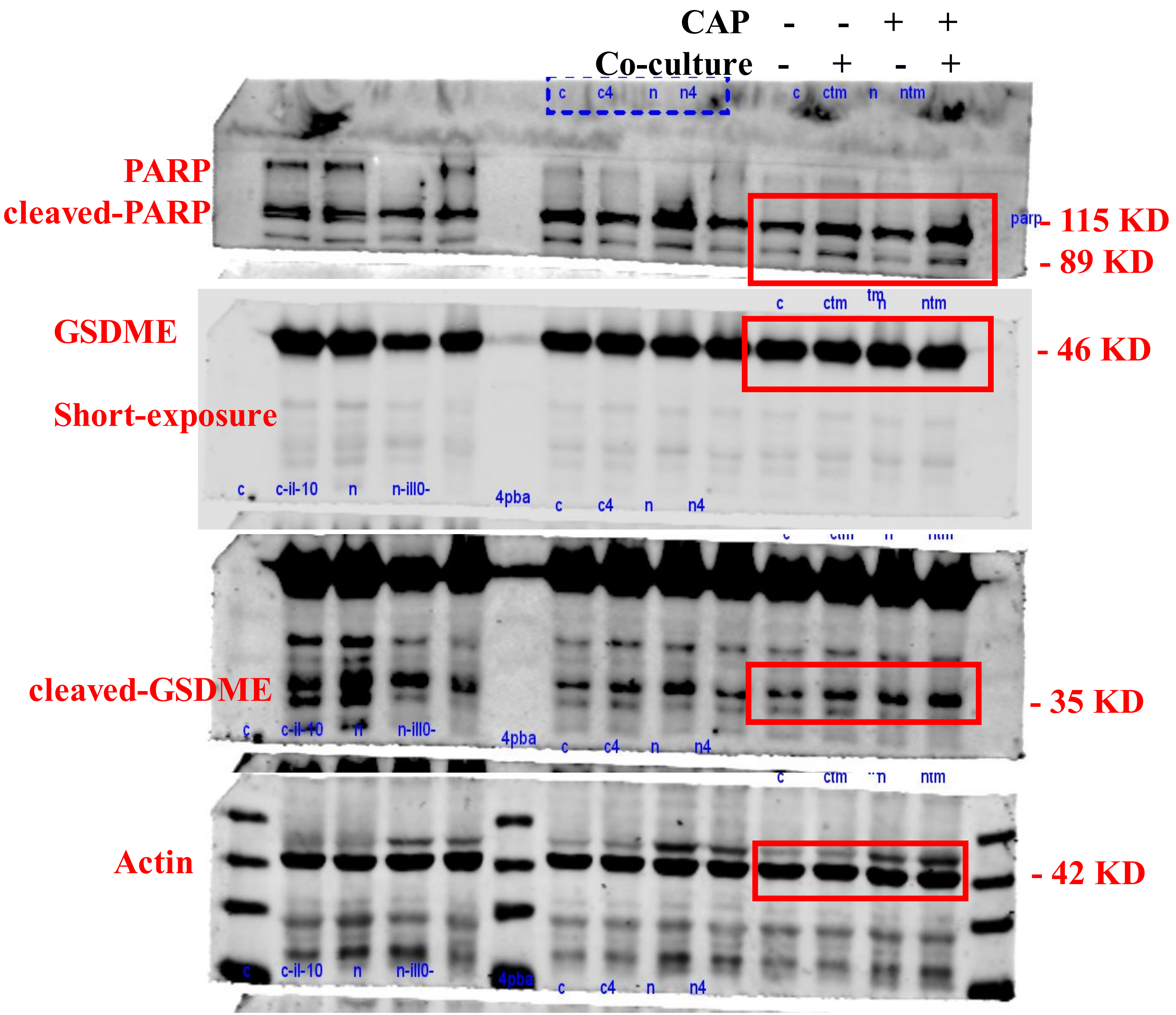

Fig 5C (Calu-1)

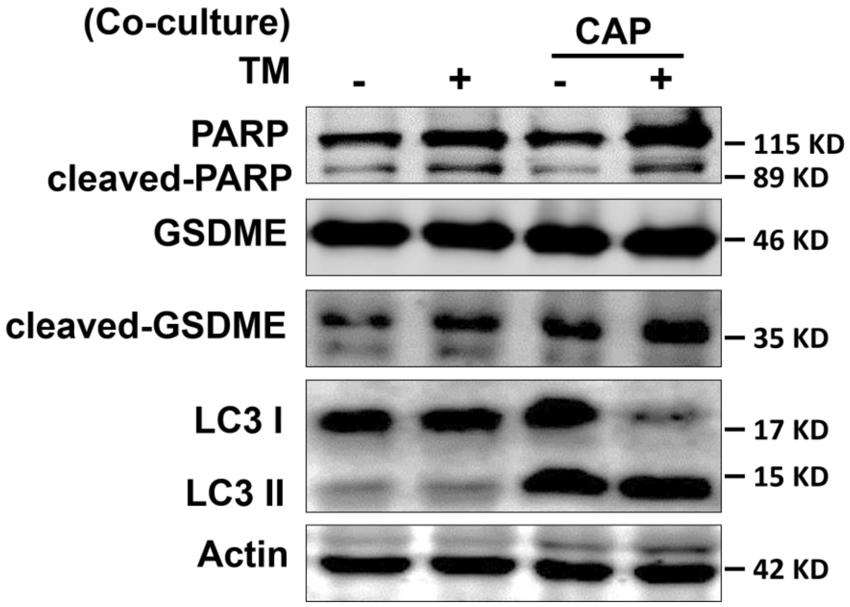

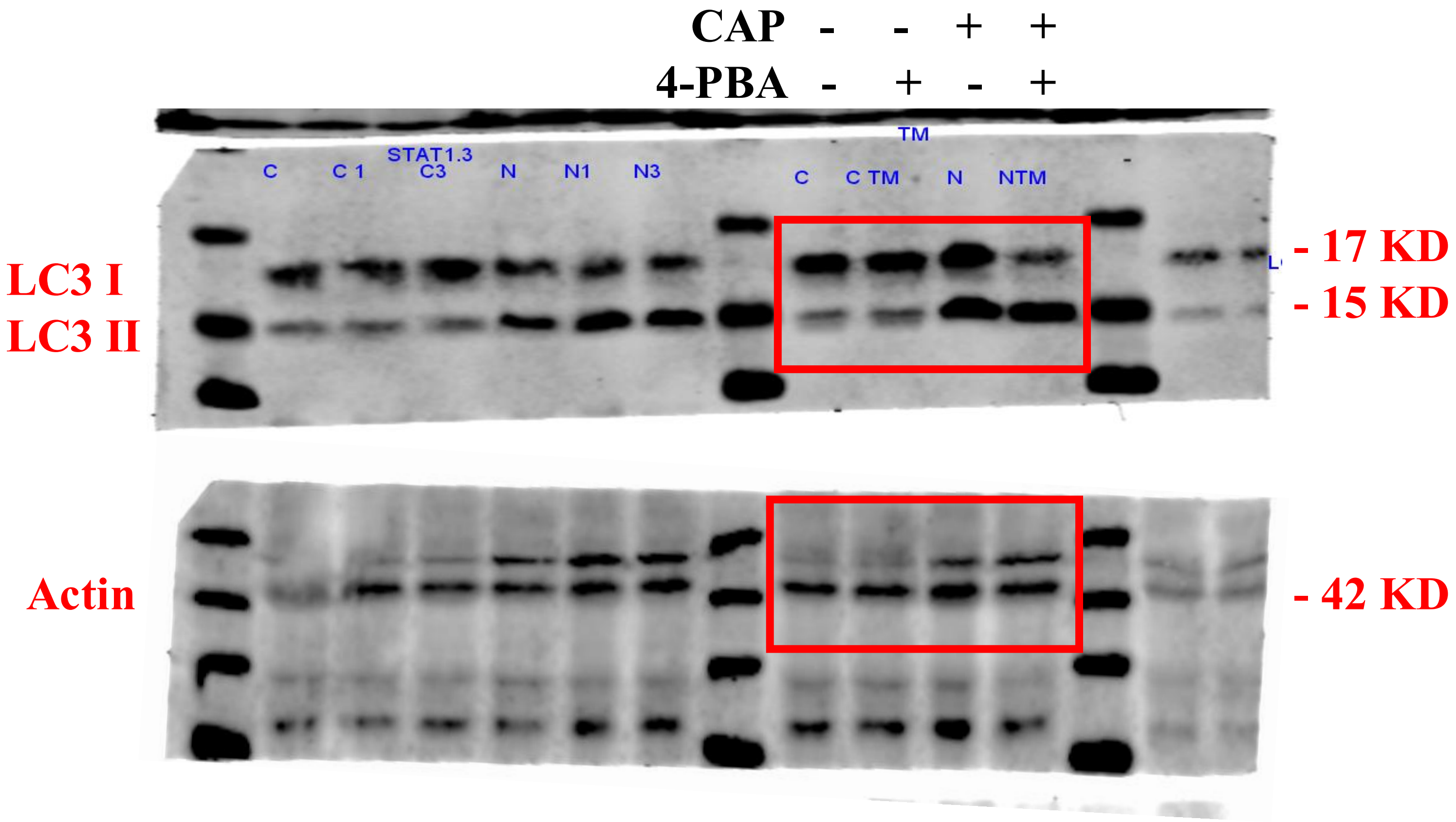

Fig 5C. The protein expression levels of PARP, Caspase9, Caspase3,GSDME, LC3 and Actin in Calu-1 cells after CAP and TM treatment.

Extended Figure 5A (Calu-1)

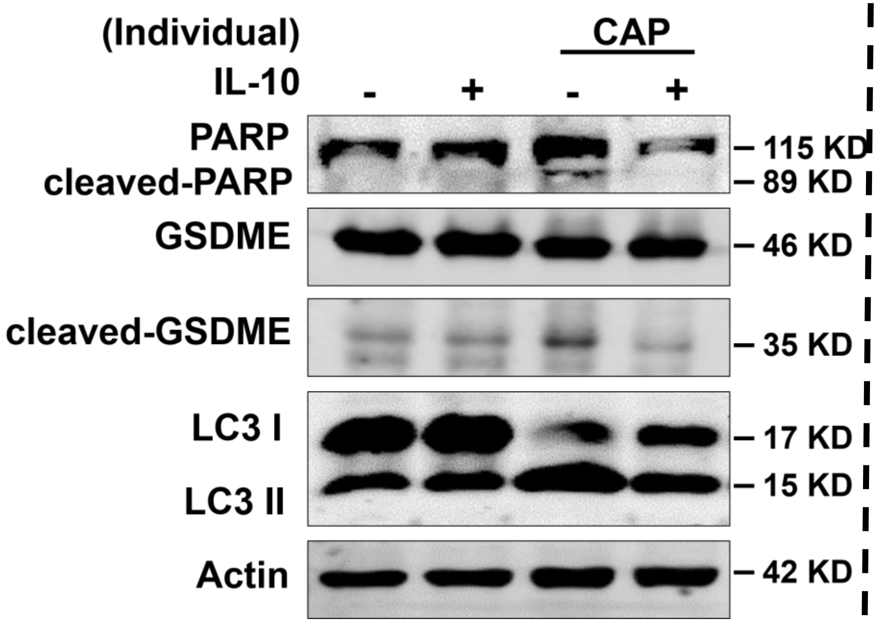

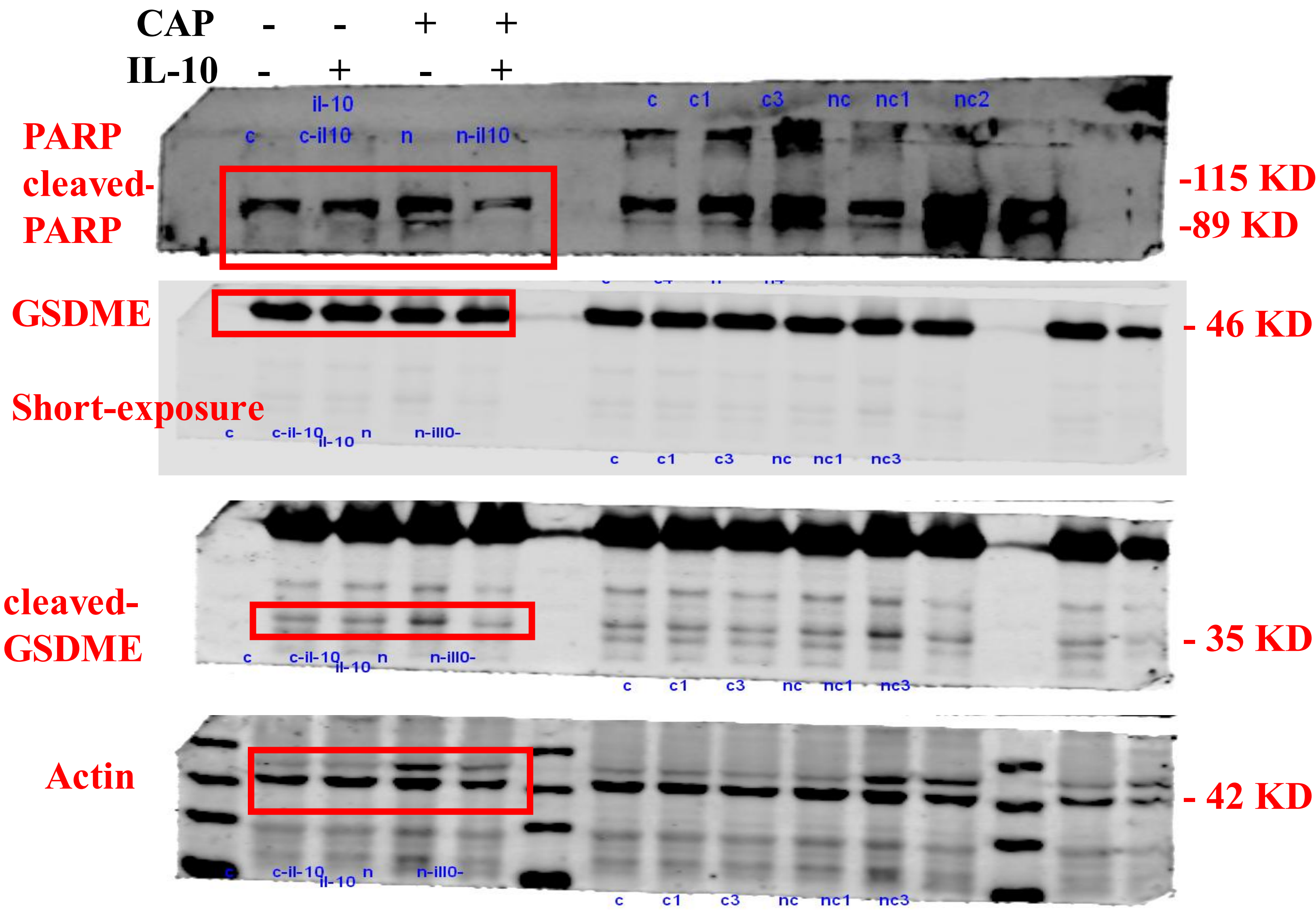

Extended Figure 5A. The protein expression levels of PARP, Caspase9, Caspase3,GSDME, LC3 and Actin in Calu-1 cells after CAP and IL-10 treatment.

Extended Figure 5A (Calu-1)

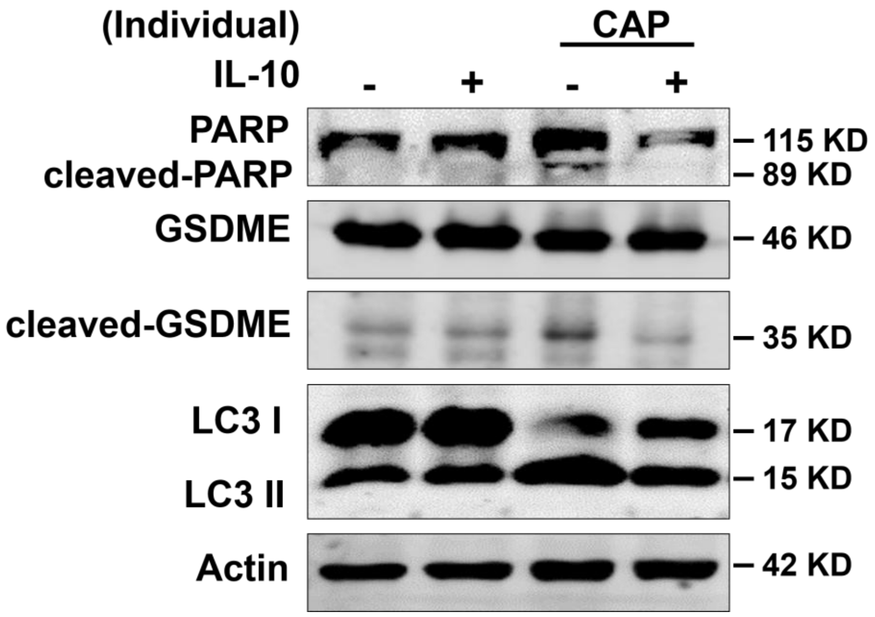

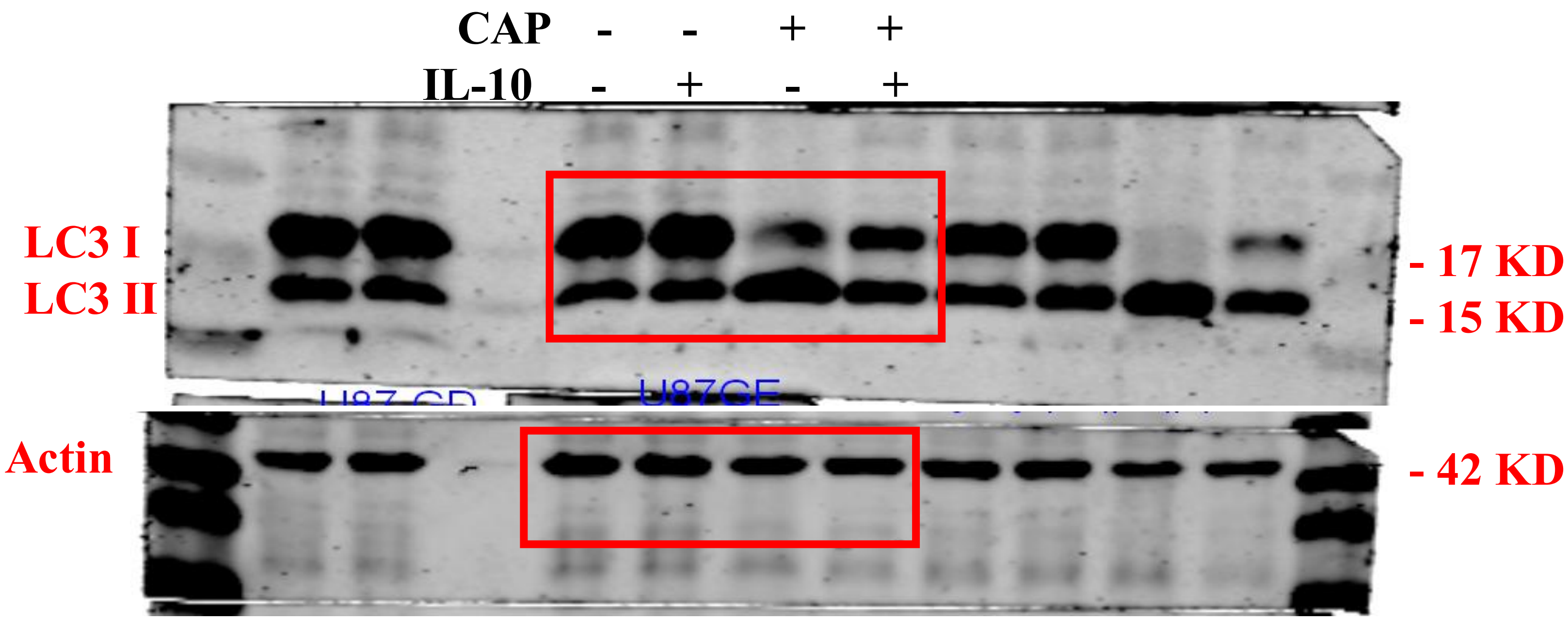

Extended Figure 5A. The protein expression levels of PARP, Caspase9, Caspase3,GSDME, LC3 and Actin in Calu-1 cells after CAP and IL-10 treatment.

Extended Figure 5B (Calu-1)

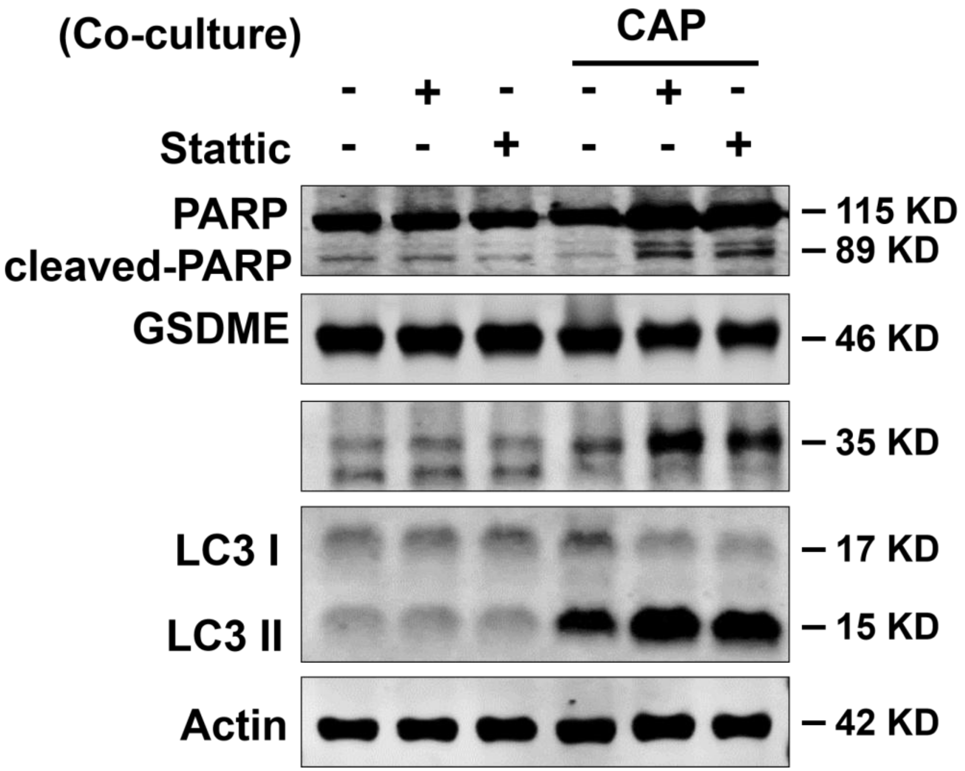

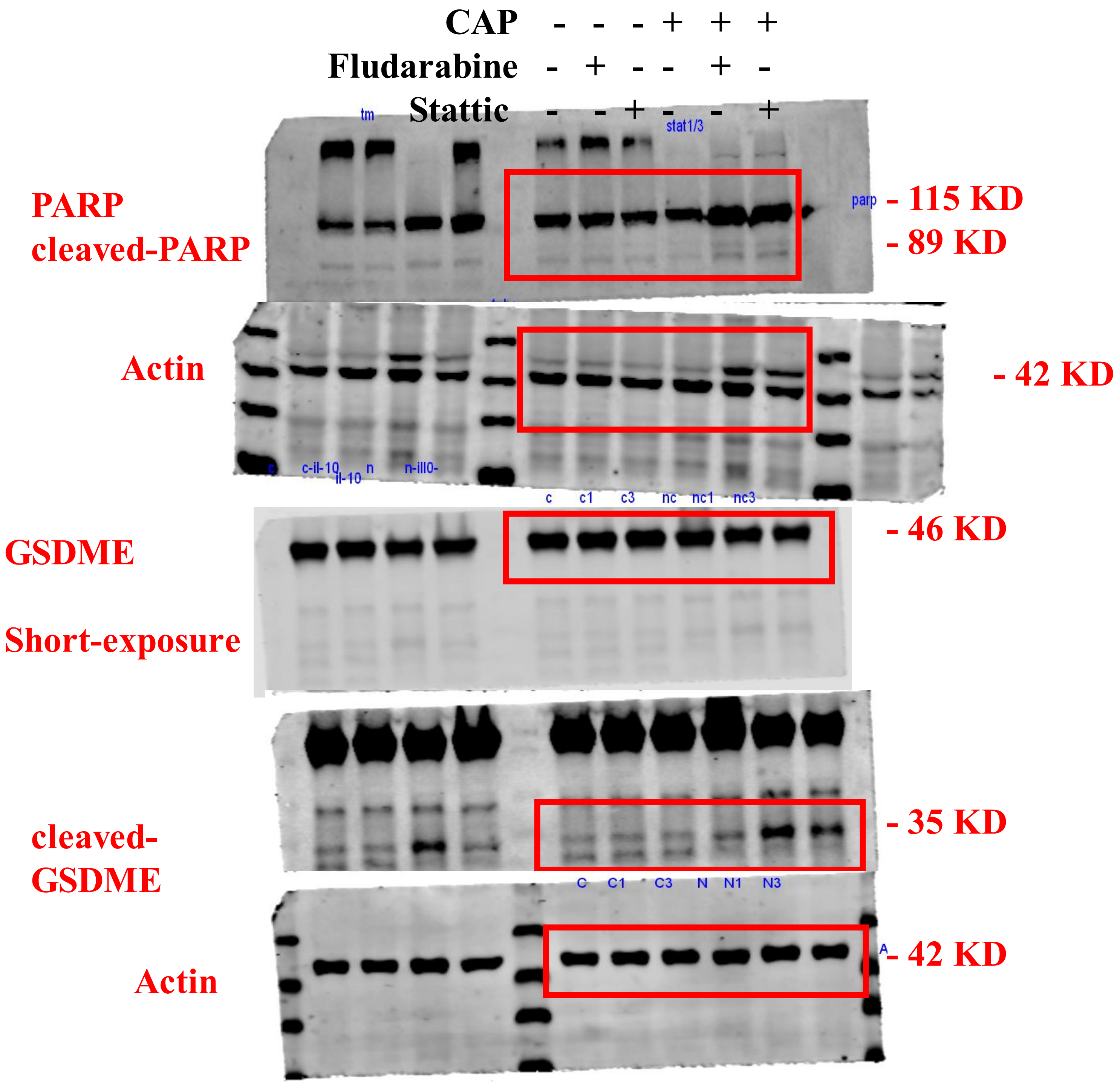

Extended Figure 5B. The protein expression levels of PARP, Caspase9, Caspase3, GSDME, LC3 and Actin in Calu-1 cells after CAP and Fludarabine, Stattic treatment.

Extended Figure 5B (Calu-1)

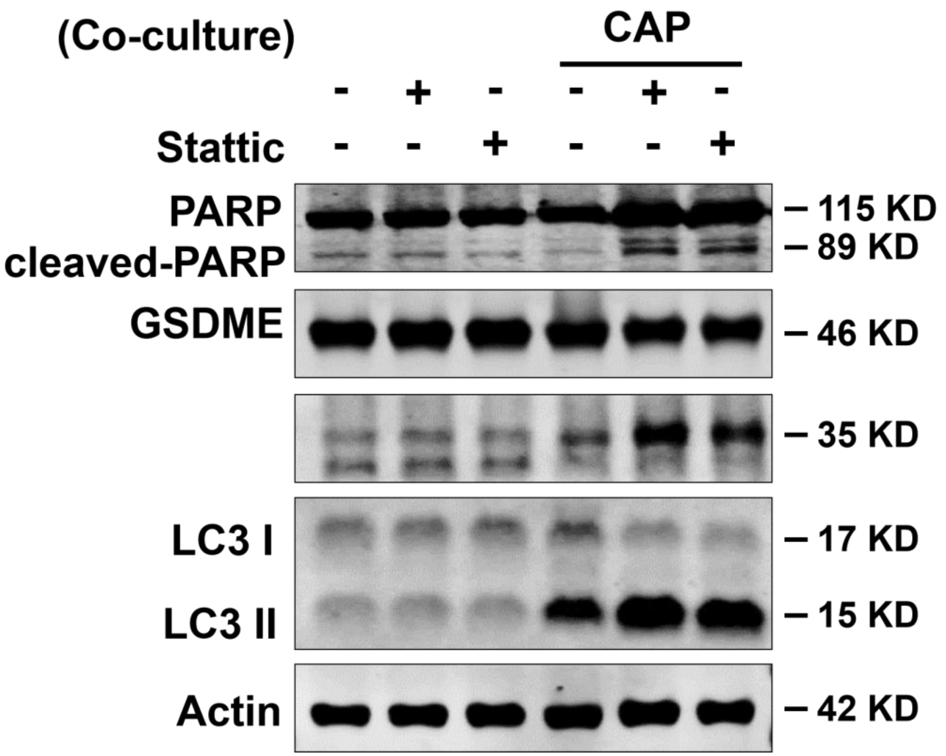

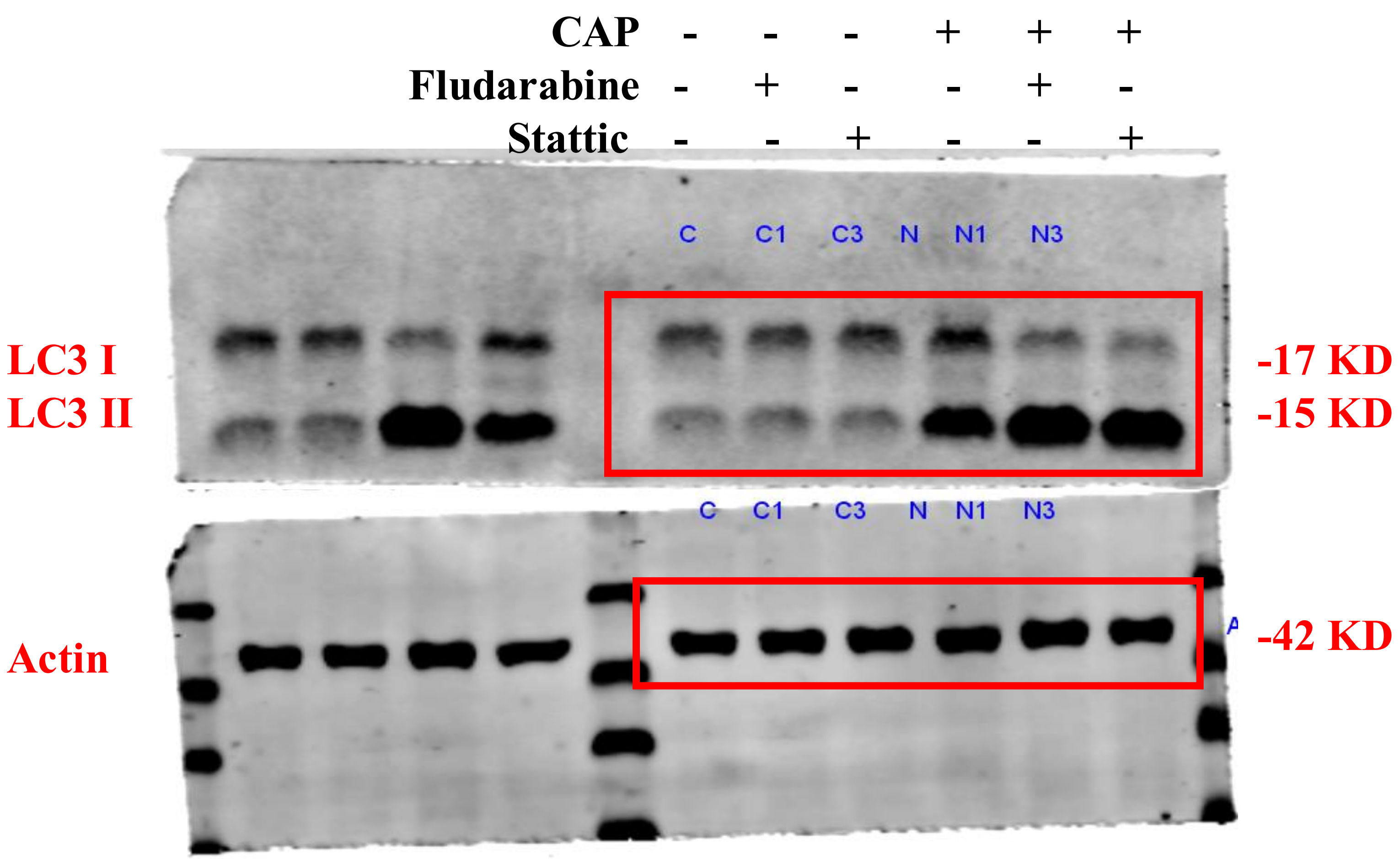

Extended Figure 5B. The protein expression levels of PARP, Caspase9, Caspase3,GSDME, LC3 and Actin in Calu-1 cells after CAP and Fludarabine, Stattic treatment.
